# FerroEnrich: an interactive web platform for liver-focused ferroptosis index scoring, enrichment, and ferroptosis–senescence cross-talk analysis

**DOI:** 10.1093/bioadv/vbag285

**Published:** 2026-09-24

**Authors:** Munichandra Babu Tirumalasetty, Mayank Choubey, Mohammad Sarif Mohiuddin, Rashu Barua, Wahiduzzaman Md, Qing Robert Miao

**Affiliations:** Department of Foundations of Medicine, New York University Grossman Long Island School of Medicine, Mineola, NY, United States; Department of Foundations of Medicine, New York University Grossman Long Island School of Medicine, Mineola, NY, United States; Department of Foundations of Medicine, New York University Grossman Long Island School of Medicine, Mineola, NY, United States; Department of Foundations of Medicine, New York University Grossman Long Island School of Medicine, Mineola, NY, United States; Department of Foundations of Medicine, New York University Grossman Long Island School of Medicine, Mineola, NY, United States; Department of Foundations of Medicine, New York University Grossman Long Island School of Medicine, Mineola, NY, United States

## Abstract

**Motivation:**

Ferroptosis is an iron-dependent regulated cell death process involving lipid peroxidation, redox imbalance, and impaired antioxidant defense. It is increasingly relevant to metabolic liver disease and non-alcoholic steatohepatitis. Existing ferroptosis resources mainly provide gene annotations and disease associations, but they do not offer an integrated workflow for user-uploaded transcriptome data, liver-focused enrichment, dataset-level scoring, and ferroptosis-senescence cross-talk analysis.

**Results:**

We developed FerroEnrich, an open-source web platform for analyzing ferroptosis and senescence programs in human and mouse liver transcriptome datasets. FerroEnrich supports count matrix upload, metadata-guided differential expression analysis, identification of ferroptosis-prone and ferroptosis-resistant genes, dataset-level ferroptosis index scoring, sample-level susceptibility estimation, curated liver module enrichment, interaction network visualization, and ferroptosis-senescence cross-talk analysis. Application to human advanced non-alcoholic steatohepatitis and mouse diet-induced liver disease datasets demonstrated cross-species utility and identified conserved remodeling, including reduced ferroptosis-protective signatures and increased senescence- and fibrosis-associated modules.

**Availability and implementation:**

FerroEnrich is freely available at https://ferroenrich.shinyapps.io/ferroenrich/. Source code, documentation, and demo datasets are available at the FerroEnrich GitHub repository.

## 1 Introduction

Ferroptosis is a controlled form of cell death characterized by iron buildup, lipid peroxidation, and the failure of antioxidant defense systems (Dixon *et al*. 2012, Yang *et al*. 2014). Unlike apoptosis, necrosis, and autophagy, ferroptosis is caused by iron-dependent oxidative damage, namely impairment of glutathione-dependent lipid peroxide detoxification mediated by GPX4, as well as other protective mechanisms involving AIFM2 and GCH1 (Bersuker *et al*. 2019, Doll *et al*. 2019). Ferroptosis plays a contextual role in liver disorders. While ferroptosis activation may aid in the elimination of therapy-resistant liver cancer cells, excessive or uncontrolled ferroptosis can worsen chronic liver injury, inflammation, fibrosis, and cirrhosis (Wang *et al*. 2019, Chen *et al*. 2022). As a result, understanding when and where ferroptosis occurs in specific hepatic cell types and illness situations is crucial for liver disease research and treatment (Wu *et al*. 2021).

Ferroptosis is regulated by gene networks that control iron intake, lipid metabolism, oxidative stress, and antioxidant defense (Li *et al*. 2020). Key regulators include TFRC, which facilitates iron intake; GPX4, which detoxifies lipid peroxides; ACSL4, which enhances polyunsaturated fatty acid incorporation and increases ferroptosis sensitivity; and SLC7A11, which maintains cystine uptake and glutathione synthesis (Koppula *et al*. 2018, Tang *et al*. 2021). Transcriptional regulators including p53 and NRF2 further influence ferroptosis-related gene expression (Jiang *et al*. 2015). Ferroptosis interacts with cellular senescence, DNA damage responses, inflammatory signaling, and hepatic stellate cell activation in the liver, implying that ferroptosis and senescence work together to influence disease progression and tissue remodeling (Zhu *et al*. 2025, Stockwell 2022).

Chronic liver diseases, which include Non-alcoholic steatohepatitis (NASH) are distinguished by lipid overload, oxidative stress, mitochondrial dysfunction, inflammation, and fibrosis. These characteristics are shared with vital ferroptosis pathways such as iron dysregulation, lipid peroxide buildup, and poor antioxidant defence. In NASH condition, ferroptosis may enhance hepatic injury, inflammation, and fibro genic remodelling, emphasizing the usefulness of transcriptome- based ferroptosis assessment in liver disease datasets (Tsurusaki *et al*. 2019, Qi *et al*. 2020). Despite the growing literature on ferroptosis, systematically assessing ferroptosis enrichment and senescence-associated transcriptional programs in human liver transcriptomes and experimental illness datasets remain complex. Several key digital resources have improved the ferroptosis area. FerrDb (Zhou and Bao 2020) was first manually curated database of ferroptosis regulators and illness associations, and later updated as FerrDb V2 (Zhou *et al*. 2023), which included additional annotations of ferroptosis drivers, suppressors, markers, regulators, compounds, and ferroptosis-disease connections. More recently, FerrDb V3 (Zhou *et al*. 2026) expanded the database beyond ferroptosis annotations to include different types of controlled cell death. These resources give useful information about ferroptosis-related genes and illness associations. In parallel, FERREG (Zhou *et al*. 2024) was created as a curated database on ferroptosis-related illness occurrence, progression, treatment response, molecular mechanisms, targets, regulators, and ferroptosis-related pharmaceuticals.

However, these resources predominantly serve as knowledge bases and do not offer a complete process workflow for user-uploaded transcriptomic datasets. To address these limitations, we created FerroEnrich an integrated platform for examining transcriptional programs linked to ferroptosis and senescence in liver disease. FerroEnrich conducts differential expression analysis on RNA-seq count data that has been uploaded by the user and then maps the resulting genes to curated ferroptosis-prone and ferroptosis-resistant gene sets. It computes a dataset-level, FerroScore-weighted Ferroptosis Index Value (FIV) by combining gene-level log2 fold changes with curated evidence scores, and it estimates sample-level susceptibility with a Ferroptosis Susceptibility Index (FSI) based on pro-ferroptotic and protective module activities. The web tool also analyzes ferroptosis and senescence associated customized pathway and gene set enrichment, creates STRING-based interaction networks, and assesses connections between ferroptosis and senescence modules. Curated biological direction and observed transcriptome relationships are provided separately to prevent correlations from being misinterpreted as changes in established gene or module function. Together, these outputs give gene, pathway, network, dataset, and sample level insights into ferroptosis-associated liver transcriptomes.

## 2 Methods

### 2.1 Tool development

FerroEnrich was built as an interactive web application with the R Shiny framework. The platform was created to provide a complete workflow for liver-focused ferroptosis and senescence transcriptome research. The user interface is divided into seven main tabs: Load Data, DEG, Pathways, Enrichment, Network, Cross-talk, and Manual.

Users can use the Load Data option to upload a read count matrix and sample metadata file, or they can choose one of two built-in demo datasets. The DEG tab conducts differential expression analysis and generates a printable result table and configurable volcano plot and computes the Ferroptosis Index Value (FIV) which summarizes dataset-level ferroptosis activity based on weighted expression changes in ferroptosis-prone and ferroptosis-resistant genes. The Pathways tab lists curated ferroptosis and senescence modules. The Enrichment tab runs module-level enrichment analyses and provides normalized enrichment scores, nominal *P*-values, and corrected *P*-values. The Network tab creates gene interaction and module relationship networks. The Crosstalk tab assesses functional interactions between ferroptosis and senescence modules. The Manual tab includes user instructions and documentation.

### 2.2 Data input, validation, and demo datasets

FerroEnrich supports bulk RNA-seq raw count matrices with genes as rows and samples as columns, as well as a matching sample metadata file including sample identification and experimental group details. The interface allows users to pick the sample ID, group column, and reference level. Prior to analysis, FerroEnrich checks to see if the sample names in the count matrix and metadata match appropriately.

The current implementation makes use of a DESeq2-compatible methodology to perform raw count-based differential expression analysis. This input format was chosen since it is often used in bulk RNA-seq analysis and maintains count-based statistical modeling. Intermediate outputs can be reviewed and downloaded to improve reproducibility.

FerroEnrich also includes two built-in demonstration datasets: a human NASH F4 stage transcriptomic dataset (Demo data 1, GSE135251) (Govaere *et al*. 2020) and our lab generated 16-week NASH diet mouse liver transcriptome dataset (Demo data 2). These sample datasets allow users to evaluate the entire workflow without the need for external input files, demonstrating FerroEnrich’s applicability across human illness data and mouse experimental models.

### 2.3 Differential expression analysis

FerroEnrich analyzes differential expression using a DESeq2-based approach (Love *et al*. 2014). After the user selects the grouping variable and reference level, the application generates the design formula and compares each experimental group to the chosen baseline. Gene identities, mapped gene symbols, log2 fold change, *P*-values, corrected *P*-values, and related data are all available as CSV files.

FerroEnrich maps gene identifiers to standardized symbols and compares important genes with curated ferroptosis-prone and ferroptosis-resistant reference lists to link differential expression results to ferroptosis biology. Matched genes are annotated using FerroScore and DEG statistics. Prone genes are pro-ferroptotic regulators, while resistant genes are defensive regulators of antioxidant defense, lipid peroxide detoxification, and iron homeostasis.

The DEG tab additionally offers customisable volcanic plot visualization. Users can customize the significance measures, log2 fold-change and *P*-value cutoffs, point and label sizes, axis text size, and number of labeled top genes. Custom gene lists can also be uploaded or pasted to highlight certain ferroptosis or senescence-related genes.

### 2.4 Calculation of Ferroptosis Index Value

FerroEnrich calculates a Ferroptosis Index Value (FIV) to estimate the total ferroptosis-related transcriptional imbalance in the analyzed dataset. FIV combines differential gene expression with gene-level FerroScore values assigned to selected ferroptosis-prone and ferroptosis-resistant genes from the FerroEnrich database.

The FerroEnrich backend contains 57 curated ferroptosis-associated genes, including 32 ferroptosis-prone and 25 ferroptosis-resistant genes with FerroScores ranging from 2 to 17 and 2 to 16, respectively. FerroScore values were collected from FerrDb and supporting literature, and they represent the relative quantity of published evidence for each gene. Thus, FerroScore serves as an evidence/literature-support weighting factor and should not be construed as a direct assessment of biological relevance, functional effect magnitude, gene potency, or ferroptosis strength. Genes were classified to the prone or resistant module based on the majority ferroptosis-promoting or ferroptosis-protective data, with the understanding that some genes may have context-dependent effects across different biological models. Table S1, Supplementary Table S1, available at supplementary material  *Bioinformatics Advances* online showed detailed gene-level documentation, such as module assignment, FerroScore, liver disease or experimental context, representative evidence, confidence grade, and supporting references.

The weighted expression score for each matched ferroptosis-associated gene was determined as follows:


$$W_{i}=\log_{2}FC_{i}\times \mathrm{FerroScor}e_{i}$$

where $W_{i}$ represents the weighted expression score of gene *i*, $\log_{2}FC_{i}$ represents the log2 fold change of gene *i* between the comparison and reference groups, and $\mathrm{FerroScor}e_{i}\;$ represents the ferroptosis relevance score assigned to gene *i* in the backend database.

Weighted scores were calculated separately for ferroptosis-prone and ferroptosis-resistant genes. The total prone score was calculated as:


$$S_{p}=\sum_{i\in P}W_{i}$$

where P represents the set of matched ferroptosis-prone genes. The total resistant score was calculated as:


$$S_{r}=\sum_{i\in R}W_{i}$$

where R represents the set of matched ferroptosis-resistant genes.

The raw FIV was then calculated as the difference between the total prone and resistant scores:


$$FIV_{\mathrm{raw}}=S_{p}-S_{r}$$

This score represents the net balance between ferroptosis-promoting and ferroptosis-protective transcriptional signals in the analyzed dataset.

To standardize the index across samples, comparisons, and datasets, the raw FIV was rescaled to a 0–5 range using min–max normalization:


$$FIV_{\mathrm{scaled}}=\frac{FIV_{\mathrm{raw}}-\min\;(F)}{\max\;(F)-\min\;(F)}\times 5$$

where $FIV_{\mathrm{scaled}}$ represents the normalized Ferroptosis Index Value, $FIV_{\mathrm{raw}}$ represents the raw index value for the analyzed comparison, min⁡ (F) and max ⁡(F) represent the minimum and maximum raw FIV values used for scaling.

The scaled FIV was then classified into five discrete interpretive categories: Normal (<2.50), Mild (2.50–2.99), Moderate (3.00–3.49), High (3.50–4.49), and Severe (≥4.50). The final scaled FIV and its corresponding category were visualized using a donut plot, providing an intuitive summary of dataset-level ferroptosis activity.

Because FIV is based on transcriptome data and curated gene weights, it should be viewed as a hypothesis-generating ferroptosis-associated transcriptional index rather than a direct experimental measurement of lipid ROS, iron buildup, GPX4 protein activity, or ferroptotic cell death. To assess the scoring framework’s resilience, sensitivity analysis was undertaken with alternative FIV formulations and FerroScore perturbation.

### 2.5 Curated pathway module construction

FerroEnrich incorporates two curated backend pathway libraries representing liver ferroptosis and liver senescence programs. These collections were created to capture the key transcriptional pathways involved in ferroptosis regulation, oxidative and metabolic stress, senescence activation, inflammatory signaling, tissue remodeling, and liver fibrosis. Figures 3A and B provide an overview of the module structure.

For pathway-level analysis, gene identifiers were standardized by changing gene symbols to uppercase or deleting version numbers from Ensembl IDs when appropriate. Users set the significance metric, *P*-value cutoff, and absolute log2 fold-change threshold. Significant genes were then split into upregulated and downregulated groups:


$$G_{up}=\{g:log_{2}FC_{g}>0,\;p_{g}<\alpha \}$$

$$G_{\mathrm{down}}=\{g:log_{2}FC_{g}<0,\;p_{g}<\alpha \}$$

where $p_{g}$ represents the selected significance value and α represents the user-defined cutoff.

For each curated module, FerroEnrich tested whether the overlap between significant genes and module genes was greater than expected by chance:

$\mathrm{Overla}p_{m}=G_{\mathrm{sig}}\cap G_{m}$

where $G_{\mathrm{sig}}$ represents the significant upregulated or downregulated gene set and $G_{m}$ represents genes in module m. Module enrichment was assessed using Fisher’s exact test, followed by Benjamini–Hochberg correction for multiple testing.

### 2.6 Gene set enrichment analysis

FerroEnrich uses gene set enrichment analysis (GSEA) to assess the pathway-level activity of curated liver ferroptosis and senescence modules (Subramanian *et al*. 2005). Gene set definitions are provided in TERM2GENE format, derived from GMT files or internally vetted backend modules that have been transformed into TERM2GENE tables. Gene identifiers are standardized by removing Ensembl version numbers and making gene symbols uppercase.

Differential expression data is used to build a ranked gene list. When the DESeq2 test statistic is available, it is utilized for ranking. Alternatively, genes are ranked using a signed significance score.


$$\mathrm{Ran}k_{i}=\mathrm{sign}(\log_{2}FC_{i})\times -\log_{10}(p_{i})$$

where $p_{i}$ represents the selected *P*-value or adjusted *P*-value. Genes with missing values or non-finite statistics are removed, and duplicate gene symbols are collapsed by retaining the entry with the largest absolute ranking statistic.

GSEA is carried out utilizing the clusterProfiler package with curated ferroptosis or senescence modules. Gene sets containing fewer than five or more than 500 genes are excluded (Yu *et al*. 2012, Xu *et al*. 2024). The results contain the module name, gene set size, enrichment score, normalized enrichment score (NES), *P*-value, adjusted *P*-value, *q*-value, rank, leading-edge information, core enrichment genes, and dataset.

For display, FerroEnrich divides enriched modules into positively enriched/upregulated and negatively enriched/downregulated modules based on NES direction. Users can show the top enriched modules as dot plots, bar plots, or lollipop plots. In these figures, the x-axis indicates the number of core enrichment genes; the y-axis shows enriched modules; and the color scale represents $-\log_{10}(\mathrm{pvalue})$ or $-\log_{10}(\mathrm{Padjusted})\;$All enrichment tables and plots can be downloaded for downstream analysis and publication-ready reporting.

### 2.7 STRING-based network design and visualization

The FerroEnrich Network module displays protein-protein interaction linkages between genes from specific liver ferroptosis or senescence modules. Following differential expression analysis, genes from the chosen module were compared to the DEG table to obtain log2 fold-change values:


$$G_{\mathrm{matched}}=G_{\mathrm{module}}\cap \mathrm{GDEG}$$

where $G_{\mathrm{module}}$ represents genes in the selected module and $G_{\mathrm{DEG}}$ represents genes detected in the differential expression table.

The STRINGdb R package was employed to standardize and map matched genes to STRING protein identifiers. The taxonomy identities 9606 and 10090 were used to map the genes of humans and mice (Szklarczyk *et al*. 2019, 2021, 2023), respectively. FerroEnrich obtained the STRING cumulative interaction score for every gene pair:


$$E_{ij}={\mathrm{STRING}}_{\mathrm{score}}(gi,gj)$$

 Where $g_{i}$ first gene/protein in the interaction pair, $g_{j}$ second gene/protein in the interaction pair, $E_{ij}$ STRING combined interaction score between $g_{i}$ and $g_{j}$

Interactions were filtered using a user-defined confidence threshold:

$\;E_{ij}^{\mathrm{filtered}}=E_{ij},ifE_{ij}\geq T$

where T stands for the chosen STRING confidence threshold. Only the top user-defined number of edges was shown, and interactions were prioritized by cumulative score to minimize network complexity.

Every node denotes a gene linked to ferroptosis or senescence, and every edge denotes an interaction supported by STRING. Gene expression changes are reflected in node color:whereas edge thickness is proportional to STRING interaction strength:


$$\mathrm{EdgeWidt}h_{ij}\propto E_{ij}$$

Networks were shown as interactive visNetwork graphs with hub-gene exploration, layout control, neighbor highlighting, and node selection. FerroEnrich employed an internal fallback network to preserve display in the event that STRING mapping failed or no interactions were found.

### 2.8 Ferroptosis-senescence cross-talk analysis

FerroEnrich uses module-level cross-talk analysis to assess the links between curated hepatic ferroptosis and liver senescence modules in a given dataset. Gene identifiers were normalized to gene symbols, raw counts to logCPM values, and module activity scores were derived as the mean normalized expression of discovered genes within each module


$$A_{ms}=(\frac{1}{n_{m}})\sum_{g=1}^{n_{m}}E_{g,s}$$

where $A_{m,s}$ is represents the activity score of module m, in the sample *s*, $E_{g}{,}_{s}$ is the normalized expression of gene *g*, in the sample *s* and $n_{m}$ represents the number of detected genes in that module.

For each ferroptosis module $F_{i}$ and senescence module $S_{j}$, Pearson correlation was calculated across samples:


$$r_{ij}=\mathrm{cor}(A_{F_{i}},A_{S_{j}})$$

where $r_{ij}$ denotes the dataset-specific module correlation between ferroptosis module $F_{i}$ and senescence module $S_{j}$. Positive correlations suggest coordinated module activity, while negative correlations indicate opposite activity patterns.

Each module pair also received a predefined literature-based direction score $L_{ij}$, where positive values imply ferroptosis-promoting prior interactions and negative values indicate ferroptosis-protective prior interactions. To rank module-pair relationships, crosstalk strength was determined as:


$$C_{ij}=|L_{ij}\times r_{ij}|$$

where $C_{ij}$ is cross talk strength between ferroptosis module, i and senescence module, j, $L_{ij}$ is literature-based direction score and $r_{ij}$ is data-set specific correlation.

FerroEnrich computes the mean Log2FC of detected genes within the module for the dataset-specific interpretation.


$$MFC_{m}=\mathrm{mean}(\log2FC_{g}),\;g\in m$$

FerroEnrich calculated the mean module log2 fold change as $MFC_{m}=\mathrm{mean}(\log2FC_{g}),g\in m$, and classified modules as upregulated, downregulated, or neutral using a near-zero threshold. Dataset-specific interpretation combined module role with observed ferroptosis- and senescence-module expression directions. Results were shown as a cross-talk context heatmap and network, where edge thickness indicates cross-talk strength, edge color indicates dataset context, and edge style indicates correlation direction.

The network was created using the igraph package (Csárdi and Nepusz 2006). Users can choose from several styles, including Fruchterman-Reingold, Kamada-Kawai, and Circle, then export the final network as a publication-ready PDF (Kamada and Kawai 1989, Fruchterman and Reingold 1991).

### 2.9 Ferroptosis Susceptibility Index (FSI)

The Ferroptosis Susceptibility Index (FSI) was calculated to assess ferroptosis susceptibility at the sample level. First, ferroptosis module scores were calculated for each sample using cross-talk analysis. Protective modules consisted of selected modules reflecting ferroptosis-protective action, such as CoQ10/FSP1, GCH1/BH4, NRF2/KEAP1 antioxidant response, and mitochondrial protection. All remaining ferroptosis modules were classified as pro-ferroptotic modules.

For each sample, FSI was computed as follows:

FSI = mean pro-ferroptotic module score − mean protective module score

Higher FSI values imply more pro-ferroptotic activity and increased ferroptosis susceptibility, while lower or negative FSI values suggest stronger protective activity and reduced ferroptosis susceptibility. The estimated FSI values were combined with sample metadata and presented as density plots and group-wise violin/boxplots. FSI values and charts can be exported to CSV and PDF formats.

## 3 Results

### 3.1 FerroEnrich provides an integrated workflow for liver ferroptosis–senescence analysis

FerroEnrich is a comprehensive web-based platform that evaluates ferroptosis and senescence activity at the transcriptome level in datasets related to liver disease (Fig. 1). FerroEnrich performs sample matching and differential expression analysis between certain experimental and reference groups once users supply a gene-level count matrix and corresponding sample metadata. By mapping important genes to carefully selected lists of ferroptosis-prone and ferroptosis-resistant genes, the Ferroptosis Index Value (FIV) can be computed utilizing matched genes and FerroScore values. For pathway enrichment, gene network visualization, and ferroptosis–senescence cross-talk analysis, these genes are then allocated to carefully selected liver ferroptosis and senescence modules. When combined, these workflows enable users to understand ferroptosis activity and its relationship to senescence-associated liver damage programs at the gene-, pathway-, network-, and cross-talk levels from transcriptome data.

**Figure 1:**
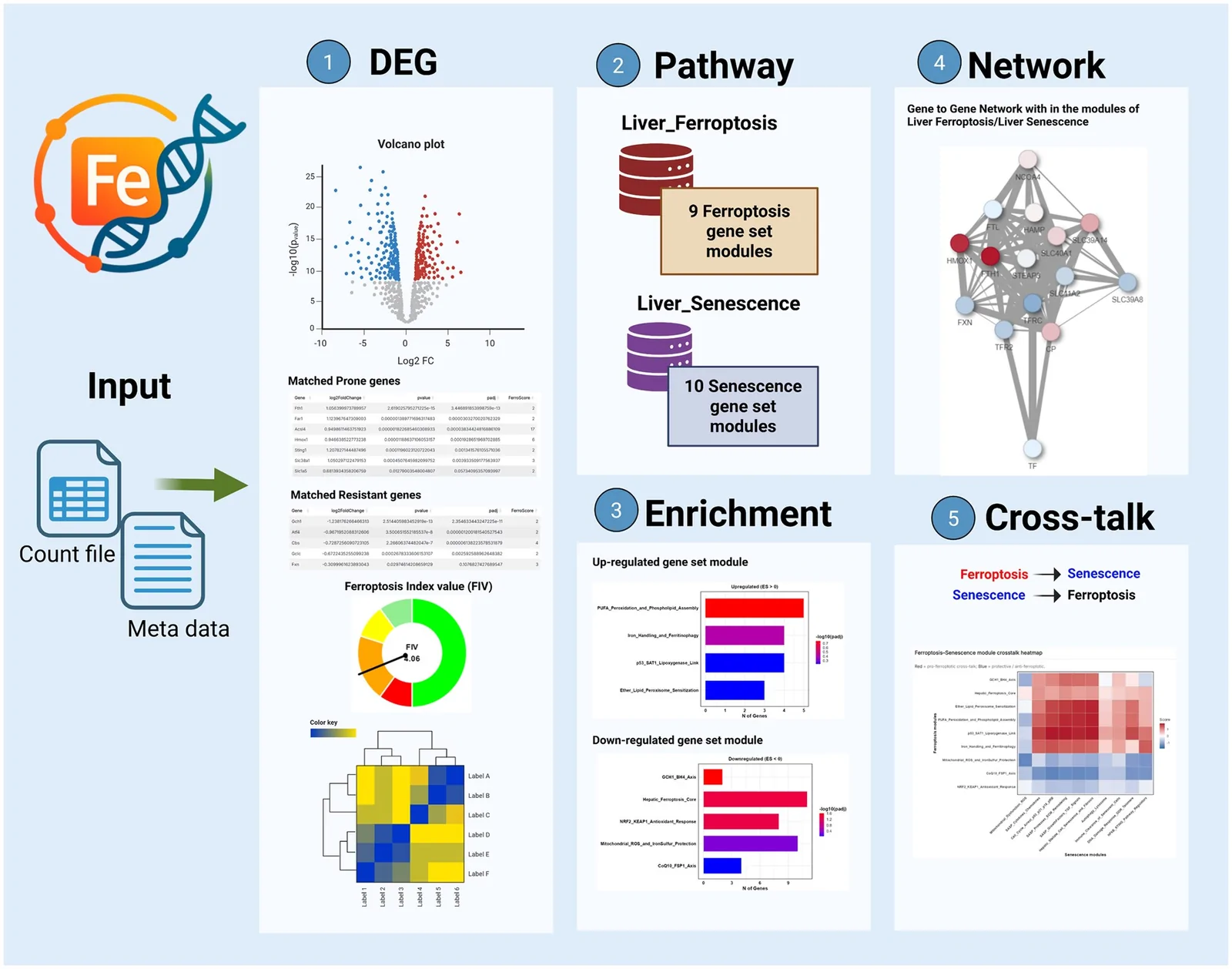
FerroEnrich workflow. The workflow starts with input of a count matrix and sample metadata. Differential expression analysis identifies significant genes and matched ferroptosis-prone and ferroptosis-resistant genes, which are used to calculate the Ferroptosis Index Value (FIV). Significant genes are then mapped to curated liver ferroptosis and liver senescence modules, followed by module enrichment analysis, gene interaction network construction, and ferroptosis–senescence cross-talk analysis. This workflow enables integrated interpretation of differential expression, pathway activity, and regulatory interactions.

### 3.2 User interface supports demo datasets, user data upload, differential expression analysis, and FIV-based interpretation

FerroEnrich was created to analyze transcriptome data submitted by users as well as built-in demo datasets. Users can upload an RNA-seq count matrix with corresponding metadata or choose Demo Data 1 or Demo Data 2, as illustrated in Fig. 2A. Human NASH F4 samples and healthy controls are included in Demo Data 1, whereas our lab-generated mouse NASH model comparing 16-week NASH diet-treated and chow-fed mice is included in Demo Data 2. Metadata files should include sample identification and group information, and uploaded count files should have genes as rows and samples as columns.

**Figure 2:**
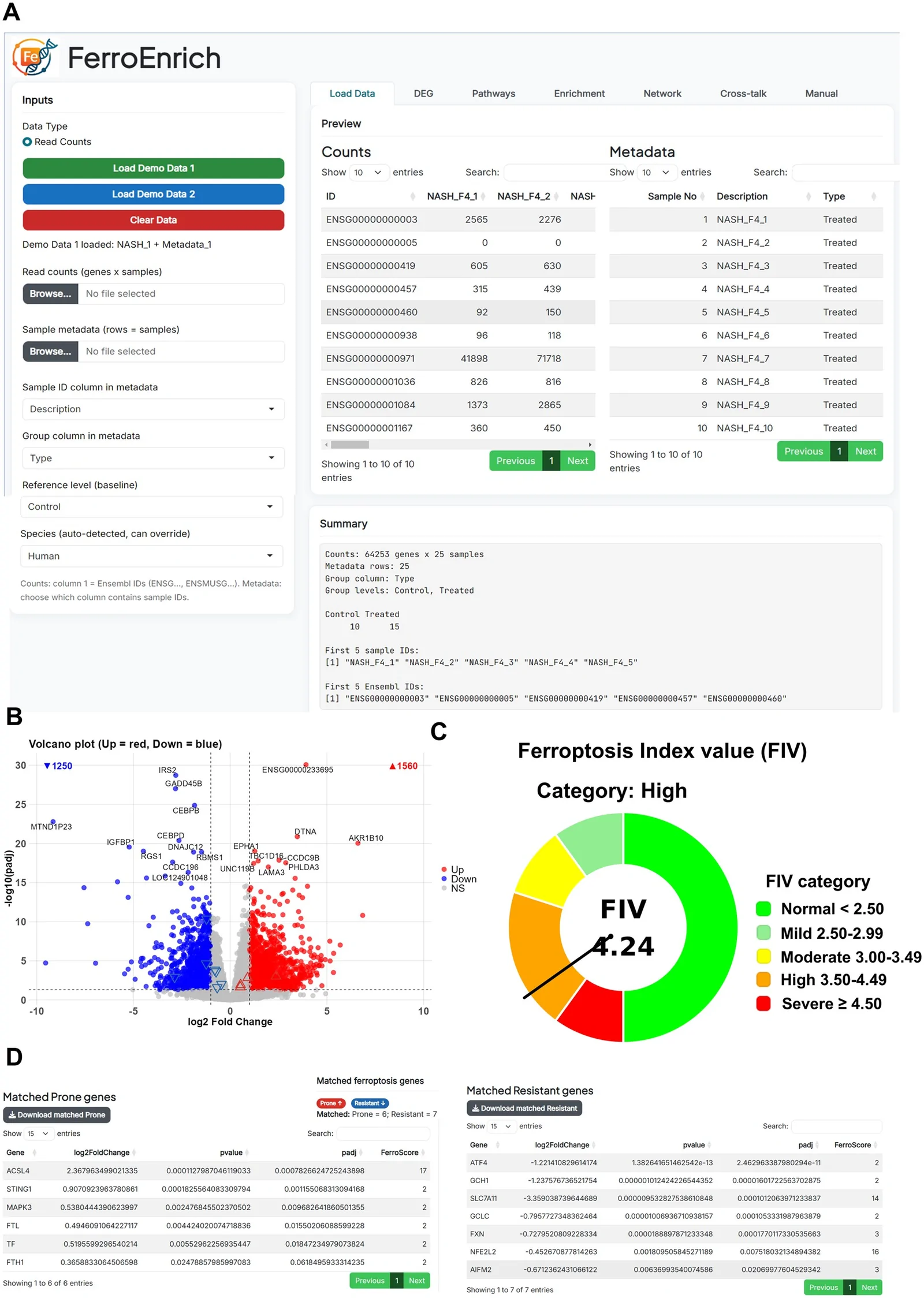
FerroEnrich user interface and ferroptosis index analysis. (A) FerroEnrich input and preview interface showing built-in demo data selection, uploaded count matrix/metadata options, sample grouping, reference group selection, and data summary. (B) Volcano plot displaying differentially expressed genes, with upregulated genes shown in red, downregulated genes in blue, and non-significant genes in gray. (C) Ferroptosis Index Value (FIV) donut plot showing the overall ferroptosis category; the representative human NASH F4 dataset was classified as High with an FIV of 4.24. (D) Matched ferroptosis-prone and ferroptosis-resistant gene tables showing log2 fold change, *P* value, adjusted p value, and FerroScore for genes detected in the input dataset.

After loading, FerroEnrich presents the count matrix, metadata, sample grouping, reference level, and dataset summary, allowing users to ensure proper matching before proceeding with the analysis. The Clear Data option lets users reset the dataset or move between demo datasets. FerroEnrich uses the specified reference group to conduct differential expression analysis and generate a volcano graphic (Fig. 2B). In Demo Data 1, NASH F4 samples were compared to healthy controls, with upregulated, downregulated, and non-significant genes represented in red, blue, and grey, respectively. This output gives a concise summary of disease-related transcriptional alterations.

FerroEnrich then matches significant genes to backend ferroptosis-prone and ferroptosis-resistant gene lists, calculating the Ferroptosis Index Value (FIV) (Fig. 2C). In the human NASH F4 demo dataset, the FIV was 4.24, indicating High ferroptosis. The donut plot is an accessible visual overview of the general ferroptosis tendency in the investigated dataset. The platform also generates matched prone and resistant gene tables (Fig. 2D). These tables show the ferroptosis-associated genes found in the dataset, along with their log2 fold change, *P*-value, corrected *P*-value, and FerroScore. This enables users to determine the specific pro-ferroptotic and protective genes that contribute to the overall FIV score. A similar analysis was done using Demo Data 2, comparing mouse NASH diet samples to chow-fed controls (Fig. S1A-C, Supplementary Table S1, available at supplementary material  *Bioinformatics Advances* online). This dataset also indicated a High ferroptosis category, with a FIV of 4.06, and produced matched prone and resistant gene tables summarizing the ferroptosis-associated score contributors. These results show that FerroEnrich can analyze built-in human and mouse liver disease datasets as well as user-uploaded count and metadata files, allowing for a complete workflow from data input to differential expression analysis, FIV scoring, and gene-level ferroptosis interpretation.

### 3.3 FerroEnrich defines curated liver ferroptosis and senescence modules for cross-species pathway interpretation

FerroEnrich has two curated backend pathway collections: a liver ferroptosis module library and a liver senescence module library. The ferroptosis library includes nine biologically defined modules that address core ferroptosis regulation, PUFA peroxidation, ether lipid/peroxisome sensitization, iron handling and ferritinophagy, CoQ10/FSP1 signaling, GCH1/BH4 defense, NRF2/KEAP1 antioxidant response, mitochondrial ROS and iron-sulfur protection, and p53/SAT1 lipoxygenase signaling (Fig. 3A). The senescence library includes 10 modules that address cell-cycle arrest, DNA damage response, SASP signaling, extracellular matrix remodeling, mitochondrial failure, autophagy/lysosome pathways, NF-κB/STING signaling, immunological clearance, and hepatic stellate cell senescence/fibrosis (Fig. 3B). These modules enable FerroEnrich to measure ferroptosis and senescence activity at the pathway level rather than just individual genes. To demonstrate cross-species applicability, we applied these modules to two built-in datasets: a human NASH F4 dataset and a mouse NASH diet dataset.

**Figure 3:**
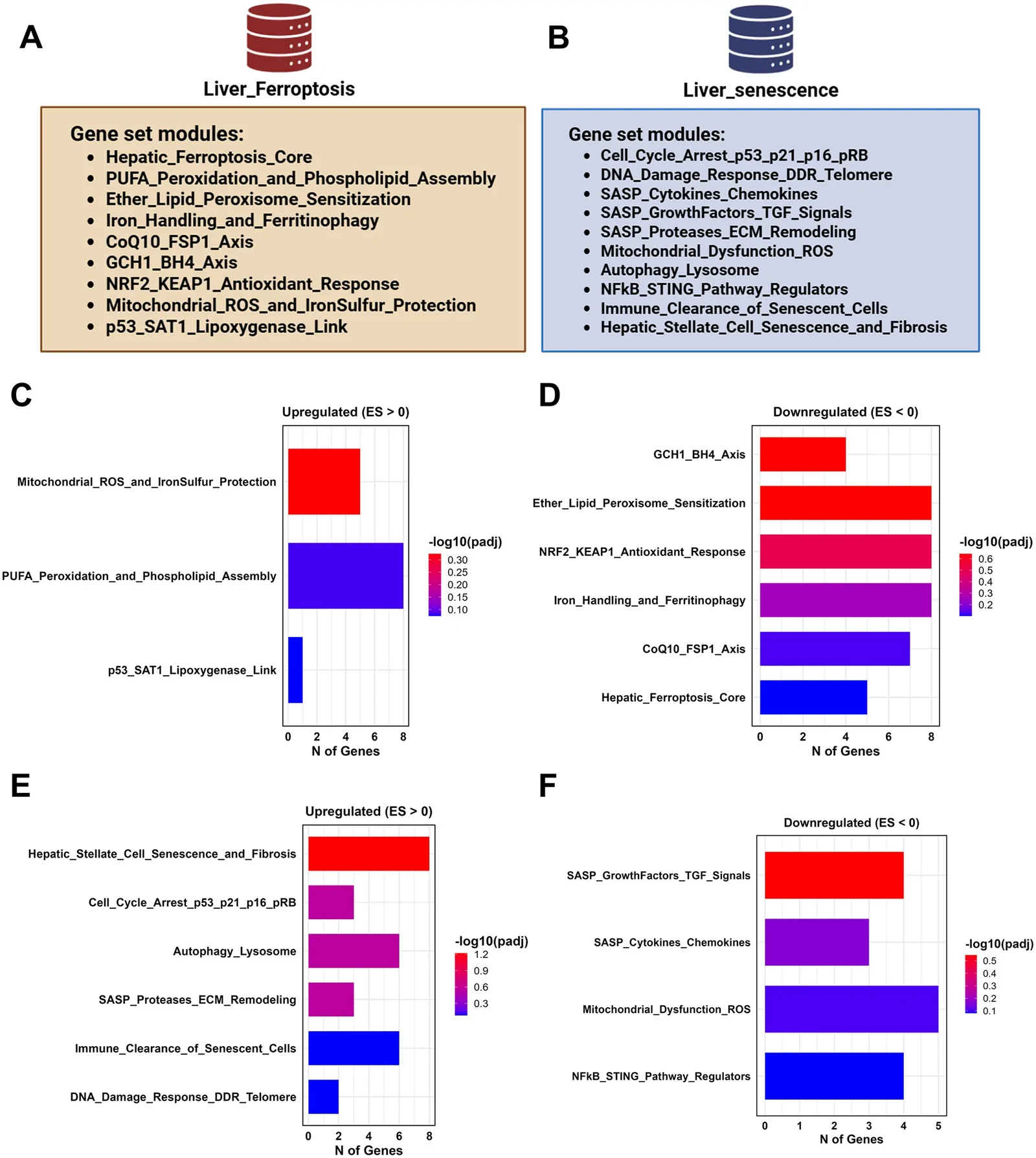
Curated liver ferroptosis and senescence modules and enrichment analysis in the human NASH F4 dataset. (A)Curated liver ferroptosis module library incorporated into FerroEnrich. Modules include hepatic ferroptosis core, PUFA peroxidation and phospholipid assembly, ether lipid/peroxisome sensitization, iron handling and ferritinophagy, CoQ10/FSP1 axis, GCH1/BH4 axis, NRF2/KEAP1 antioxidant response, mitochondrial ROS/iron–sulfur protection, and p53/SAT1 lipoxygenase signaling. (B) Curated liver senescence module library, including cell-cycle arrest, DNA damage response/telomere biology, SASP cytokines/chemokines, SASP growth factor/TGF signaling, SASP proteases/ECM remodeling, mitochondrial dysfunction/ROS, autophagy/lysosome, NF-κB/STING signaling, immune clearance of senescent cells, and hepatic stellate cell senescence/fibrosis. (C) Upregulated liver ferroptosis modules in human NASH F4 liver, (D) Downregulated liver ferroptosis modules in human NASH F4 liver. (e) Upregulated liver senescence modules in human NASH F4 liver. (f) Downregulated liver senescence modules in human NASH F4 liver, Bar length represents the number of core enrichment genes, and color represents − log10 adjusted *P*-value.

### 3.4 Human NASH F4 dataset shows altered ferroptosis defense and senescence-associated fibrotic remodeling

Using the human NASH F4 demo dataset, FerroEnrich performed gene set enrichment analysis across liver ferroptosis and liver senescence modules. The complete human enrichment results were summarized in Table 1. The human NASH F4 module-level results are interpreted as exploratory enrichment trends, even though several modules showed directional enrichment; none reached statistical significance after Benjamini–Hochberg correction (FDR < 0.05). Several protective or regulatory liver ferroptosis modules were shown to be enriched negatively in the human NASH F4 liver. The GCH1_BH4_Axis had a negative enrichment (NES = −1.53), with core enrichment genes such as QDPR, SPR, PTS, and GCH1. The Ether_Lipid_Peroxisome_Sensitization module has a negative enrichment (NES = −1.56) and included core genes such as PEX19, AGPS, ACBD5, ACOX1, FAR1, ACAA1, PEX7, and PECR. Antioxidant-related genes such as TXN, GLRX2, TXNIP, NFE2L2, GLRX, PRDX6, GCLC, and SOD1 contributed to the negative enrichment of the NRF2_KEAP1_Antioxidant_Response module (NES = −1.37).

**Table 1:** Gene set enrichment analysis of liver ferroptosis and senescence modules in the human NASH F4 dataset.

Module	setSize	Enrichment score	NES	*P*-value	Adjusted *P*-value	Rank	Core enrichment genes	Dataset category
**GCH1_BH4_Axis**	5	−0.75	−1.53	0.0506	0.2277	4352	QDPR/SPR/PTS/GCH1	Liver_ferroptosis
**Ether_Lipid_Peroxisome_Sensitization**	18	−0.52	−1.56	0.0468	0.2277	4529	PEX19/AGPS/ACBD5/ACOX1/FAR1/ACAA1/PEX7/PECR	Liver_ferroptosis
**NRF2_KEAP1_Antioxidant_Response**	17	−0.46	−1.37	0.1035	0.3107	3895	TXN/GLRX2/TXNIP/NFE2L2/GLRX/PRDX6/GCLC/SOD1	Liver_ferroptosis
**Mitochondrial_ROS_and_IronSulfur_Protection**	15	0.43	1.22	0.2108	0.4743	3070	FDXR/ACO2/NDUFS2/DLST/UQCRC2	Liver_ferroptosis
**Iron_Handling_and_Ferritinophagy**	17	−0.37	−1.10	0.3087	0.5557	4346	SLC40A1/HAMP/CP/SFXN2/SFXN1/SLC11A2/SLC39A8/FXN	Liver_ferroptosis
**CoQ10_FSP1_Axis**	14	−0.34	−0.96	0.4959	0.7439	5485	COQ2/COQ3/COQ10B/AIFM2/DHODH/COQ7/COQ5	Liver_ferroptosis
**Hepatic_Ferroptosis_Core**	18	−0.29	−0.89	0.6156	0.7916	1434	IDH1/PRDX6/SLC7A11/SLC38A2/GCLC	Liver_ferroptosis
**PUFA_Peroxidation_and_Phospholipid_Assembly**	26	0.25	0.83	0.7326	0.8242	4163	FADS2/FADS1/ACSL4/POR/CYP4F2/ELOVL5/MBOAT7/ALOX15B	Liver_ferroptosis
**p53_SAT1_Lipoxygenase_Link**	9	0.29	0.71	0.8430	0.8430	393	CDKN1A	Liver_ferroptosis
**Hepatic_Stellate_Cell_Senescence_and_Fibrosis**	15	0.63	1.80	0.0060	0.0605	4929	LAMA2/COL1A1/COL3A1/COL1A2/PDGFRB/LAMB1/LOXL2/TAGLN	Liver_senescencece
**Cell_Cycle_Arrest_p53_p21_p16_pRB**	13	0.52	1.42	0.0868	0.2851	1062	CCND1/CDKN1A/E2F1	Liver_senescencece
**Autophagy_Lysosome**	15	0.46	1.33	0.1425	0.2851	2370	SQSTM1/ATG16L1/CTSD/ATG7/LAMP1/BECN1	Liver_senescencece
**SASP_Proteases_ECM_Remodeling**	10	0.53	1.32	0.1261	0.2851	585	MMP14/COL1A1/COL3A1	Liver_senescencece
**SASP_GrowthFactors_TGF_Signals**	10	−0.53	−1.36	0.1115	0.2851	2623	INHBA/IGFBP3/AREG/FGF2	Liver_senescencece
**SASP_Cytokines_Chemokines**	11	−0.40	−1.06	0.4107	0.6845	1455	CXCL3/IL6/CXCL2	Liver_senescencece
**Mitochondrial_Dysfunction_ROS**	9	−0.37	−0.92	0.5511	0.7857	1167	PRDX5/NDUFA9/TFAM/PPARGC1A/PRDX3	Liver_senescencece
**Immune_Clearance_of_Senescent_Cells**	12	0.29	0.78	0.7450	0.8286	7722	ULBP3/MICA/CX3CL1/ULBP1/ULBP2/ICAM1	Liver_senescencece
**DNA_Damage_Response_DDR_Telomere**	11	0.30	0.77	0.7570	0.8286	2102	TP53BP1/PARP1	Liver_senescencece
**NFkB_STING_Pathway_Regulators**	10	−0.28	−0.73	0.8286	0.8286	4896	TAB2/MAP3K7/TBK1/NFKBIA	Liver_senescencece

This table summarizes enrichment results from 15 NASH F4 patients and 10 healthy controls, including module description, gene set size, enrichment score, normalized enrichment score, *P*-value, adjusted *P*-value, rank, core enrichment genes, and dataset category.

In contrast, the Mitochondrial_ROS_and_IronSulfur_Protection module had a positive enrichment tendency (NES = 1.22), as did the PUFA_Peroxidation_and_Phospholipid_Assembly module (NES = 0.83). These data imply that the human NASH F4 liver contains a combination of ferroptosis-related signals, including partial activation of mitochondrial and lipid remodeling pathways, as well as suppression of antioxidant and ferroptosis-protective programs.

The human ferroptosis enrichment plots provide an additional summary of this phenomenon. Upregulated ferroptosis modules included Mitochondrial_ROS_and_IronSulfur_Protection, PUFA_Peroxidation_and_Phospholipid_Assembly, and p53_SAT1_Lipoxygenase_Link (Fig. 3C). Downregulated ferroptosis modules include GCH1_BH4_Axis, Ether_Lipid_Peroxisome_Sensitization, NRF2_KEAP1_Antioxidant_Response, Iron_Handling_and_Ferritinophagy, CoQ10_FSP1_Axis, and Hepatic_Ferroptosis_Core (Fig. 3D).

Senescence enrichment in the human NASH F4 dataset identified strong fibrotic and remodeling characteristics. The Hepatic_Stellate_Cell_Senescence_and_Fibrosis module has the highest positive enrichment trend among the senescence modules (NES = 1.80, nominal *P* = 0.00605, adjusted *P* = 0.0605), with core enrichment genes such as LAMA2, COL1A1, COL3A1, COL1A2, PDGFRB, LAMB1, LOXL2, and TAGLN. This module had the lowest adjusted *P*-value among the examined modules, but did not meet the preset FDR < 0.05 requirement. Positively enhanced senescence modules included Cell_Cycle_Arrest_p53_p21_p16_pRB, Autophagy_Lysosome, SASP_Proteases_ECM_Remodeling, Immune_Clearance_of_Senescent_Cells, and DNA_Damage_Response_DDR_Telomere (Fig. 3E).

Several senescence modules showed negative enrichment in human NASH F4 liver, including SASP_GrowthFactors_TGF_Signals, SASP_Cytokines_Chemokines, Mitochondrial_Dysfunction_ROS, and NFkB_STING_Pathway_Regulators (Fig. 3F). The analysis reveals that advanced NASH fibrosis is characterized by high hepatic stellate cell/fibrosis-associated senescence activity, as well as altered ferroptosis defense and lipid/metabolic stress programs.

### 3.5 Mouse NASH diet dataset shows stronger ferroptosis-protective suppression and robust senescence/fibrosis enrichment

We next applied FerroEnrich to the mouse NASH diet demo dataset, which included NASH diet-treated and chow diet-treated mouse, with n = 5 per group. The complete mouse enrichment results are summarized in Table S2, Supplementary Table S1, available at supplementary material  *Bioinformatics Advances* online, and the corresponding enrichment plots are shown in Fig. S2, Supplementary Table S1, available at supplementary material  *Bioinformatics Advances* online

In the mouse NASH diet dataset, ferroptosis analysis revealed a significant negative enrichment of protective ferroptosis-associated modules. The GCH1_BH4_Axis was highly negatively enriched (NES = −1.80), with core enrichment genes GCH1 and QDPR. MDH1, GPX1, ME1, SLC3A2, GCLC, SLC38A2, GCLM, GLS2, TXNRD1, IDH1, and SLC25A1 were among the core genes in the Hepatic_Ferroptosis_Core module, which was significantly enriched negatively (NES = −1.73).

The NRF2_KEAP1_Antioxidant_Response module revealed a strong negative trend with NES = −1.65, indicating decrease of antioxidant defense in the mouse NASH diet model. Other ferroptosis-related modules that were downregulated were Mitochondrial_ROS_and_IronSulfur_Protection and CoQ10_FSP1_Axis (Fig. S2A, Supplementary Table S1, available at supplementary material  *Bioinformatics Advances* online). These findings suggest that the mouse NASH diet dataset has a significant drop in ferroptosis-protective transcriptional pathways.

In contrast, some ferroptosis-prone modules exhibited a positive enrichment trend in the mouse NASH diet dataset. These included PUFA_Peroxidation_and_Phospholipid_Assembly, Iron_Handling_and_Ferritinophagy, p53_SAT1_Lipoxygenase_Link, and Ether_Lipid_Peroxisome_Sensitization (Fig. S2B, Supplementary Table S1, available at supplementary material  *Bioinformatics Advances* online). Although not all reached adjusted significance, their positive direction indicates that lipid peroxidation, iron handling, and pro-ferroptotic stress processes have been activated.

Mouse senescence enrichment revealed a pronounced fibrotic and SASP-associated pattern. The Hepatic_Stellate_Cell_Senescence_and_Fibrosis module was significantly enriched (NES = 2.29). Enrichment genes included COL1A1, COL3A1, COL1A2, LOXL2, LAMB1, PDGFRB, ACTA2, LAMA2, and LOX. The SASP_Proteases_ECM_Remodeling module was significantly enriched (NES = 2.17), containing key genes such as MMP12, COL1A1, COL3A1, PLAU, TIMP2, and TIMP1.

The Immune_Clearance_of_Senescent_Cells module was considerably enriched (NES = 1.66), supported by genes such as VCAM1, KLRK1, CX3CL1, ULBP1, ICAM1, GZMB, and PRF1. Positively enriched senescence modules include SASP_GrowthFactors_TGF_Signals, SASP_Cytokines_Chemokines, Cell_Cycle_Arrest_p53_p21_p16_pRB, and DNA_Damage_Response_DDR_Telomere (Fig. S2D, Supplementary Table S1, available at supplementary material  *Bioinformatics Advances* online). Downregulated senescence modules included Mitochondrial_Dysfunction_ROS, Autophagy_Lysosome, and NFkB_STING_Pathway_Regulators (Fig. S2C, Supplementary Table S1, available at supplementary material  *Bioinformatics Advances* online). Overall, the mouse NASH diet dataset showed more robust and statistically significant enrichment patterns than the human dataset, notably for suppressing beneficial ferroptosis modules and activating senescence/fibrosis-associated modules.

### 3.6 Cross-species comparison supports conserved ferroptosis–senescence remodeling in NASH

A comparison of the human and mouse demo datasets revealed some common aspects of NASH-associated ferroptosis and senescence remodeling. Both datasets revealed altered ferroptosis regulation, including the suppression or negative enrichment of antioxidant/protective modules such as GCH1_BH4_Axis, NRF2_KEAP1_Antioxidant_Response, CoQ10_FSP1_Axis, and Hepatic_Ferroptosis_Core. Both datasets also revealed strong enrichment trends for ferroptosis-prone modules associated with lipid peroxidation, mitochondrial stress, iron management, and p53/SAT1 signaling.

The most consistent cross-species discovery was the presence of senescence-associated fibrotic remodeling. The Hepatic_Stellate_Cell_Senescence_and_Fibrosis module was positively enriched in both human NASH F4 and mouse NASH diet datasets, with core genes linked to extracellular matrix deposition and fibrogenesis, such as COL1A1, COL3A1, COL1A2, PDGFRB, LAMB1, and LOXL2. The SASP_Proteases_ECM_Remodelling module was likewise positively enriched in both datasets, but the signal was higher in the mouse dataset.

These findings show that FerroEnrich can detect conserved species-specific ferroptosis-senescence patterns in human and mouse liver disease datasets. The human NASH F4 dataset revealed significant fibrotic senescence and moderate ferroptosis enrichment, but the mouse NASH diet dataset revealed higher inhibition of ferroptosis-protective pathways as well as substantial activation of senescence/fibrosis and SASP remodeling processes. This cross-species application validates the use of FerroEnrich for comparing clinical liver disease samples to experimental NASH models.

### 3.7 Network analysis reveals interaction structure of ferroptosis and senescence module genes

FerroEnrich provides a network analysis tool that displays functional links between genes in the curated liver ferroptosis and liver senescence gene sets. Using the human NASH F4 demo dataset, we created interaction networks for all liver ferroptosis and senescence module genes. STRING protein-protein interaction evidence was used to create network edges, and node colors denoted the direction and level of differential expression as measured by log2 fold change.

The ferroptosis network revealed a densely coupled interaction structure between genes involved in antioxidant defense, mitochondrial redox control, iron handling, lipid peroxidation, and ferroptosis protection (Fig. 4A).

**Figure 4:**
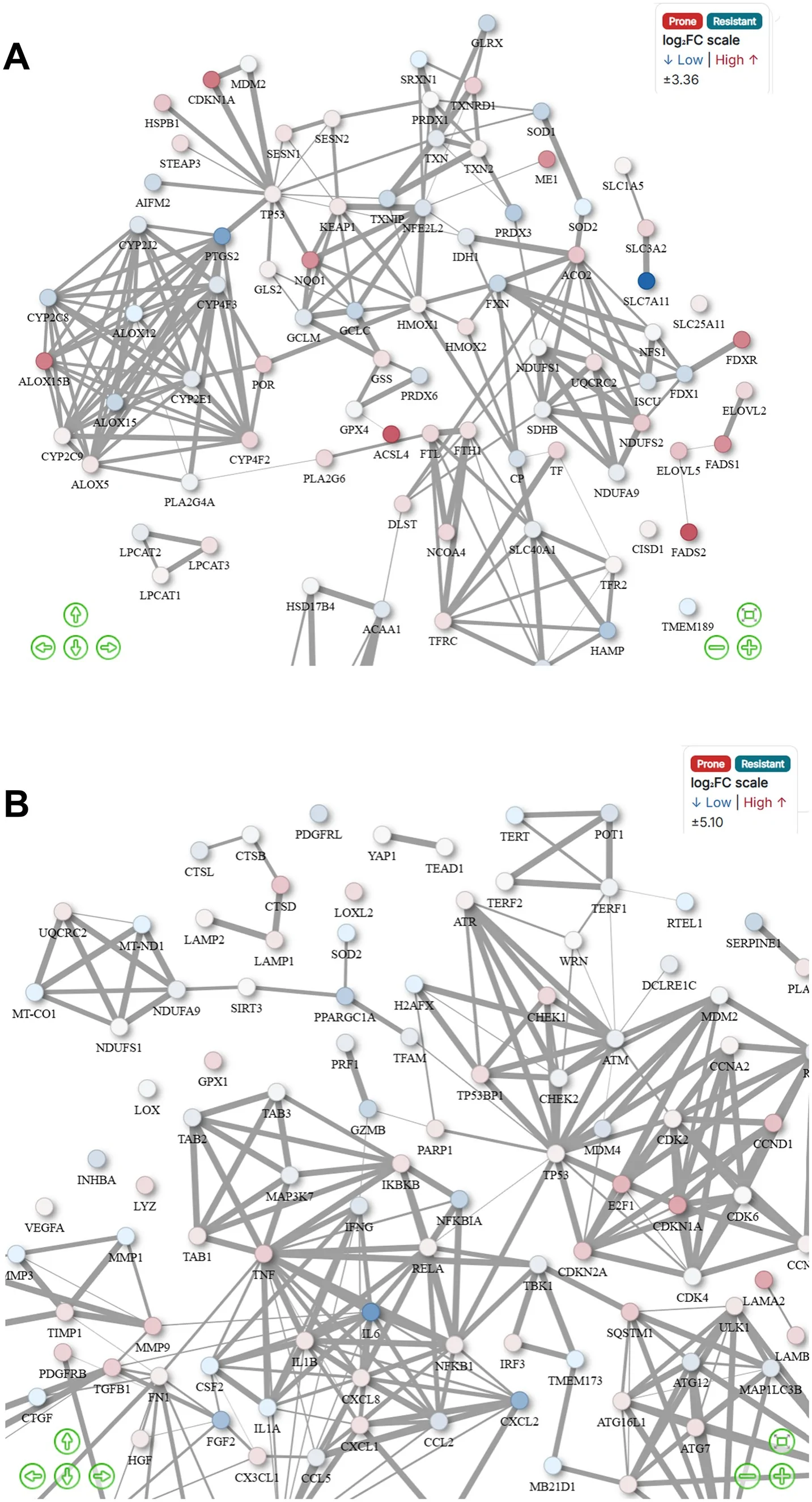
STRING-based network visualization of liver ferroptosis and senescence module genes in human NASH F4 liver. (A) Interaction network of liver ferroptosis module genes in human NASH F4 liver. (B) Interaction network of liver senescence module genes in human NASH F4 liver. Nodes represent module genes, and edges represent STRING-supported protein–protein interactions. Node color indicates log2 fold change in NASH F4 liver compared with healthy controls, with red representing upregulated genes, blue representing downregulated genes, and white representing minimal change. Edge thickness reflects interaction strength.

The network included several fundamental ferroptosis regulators, such as GPX4, SLC7A11, GCLC, GCLM, TXNRD1, NFE2L2, KEAP1, NQO1, SOD1, SOD2, FTH1, FTL, HMOX1, ACSL4, POR, ALOX15, GCH1, AIFM2, and CoQ pathway genes. The tight linkages between these genes suggest that FerroEnrich detects coordinated ferroptosis-related activities rather than isolated gene alterations.

Node coloring also enabled observation of disease-associated transcriptional direction. Red nodes represented genes that were elevated in the NASH F4 liver, while blue nodes showed genes that were downregulated in the NASH F4 liver compared to healthy controls. This display assisted in identifying transcriptionally changed genes within the ferroptosis network and demonstrated the coexistence of pro-ferroptotic and protective ferroptosis-related programs in advanced NASH.

The senescence network was tightly coupled, with cell cycle arrest, DNA damage response, inflammatory signaling, SASP activation, extracellular matrix remodeling, immunological recognition, and hepatic stellate cell activation (Fig. 4B). These interactions underscore the interconnectedness of senescence-related inflammatory and fibrotic remodeling in NASH. The same STRING-based approach was used on the mouse NASH diet dataset, which shows ferroptosis and senescence networks in Figs S3A and S3B, Supplementary Table S1, available at supplementary material  *Bioinformatics Advances* online, with node colors denoting the log2 fold-change between the NASH diet-treated and chow control groups.

These network outputs show that FerroEnrich can combine differential expression results and protein interaction evidence to create a system-level understanding of ferroptosis and senescence regulation. The ferroptosis network emphasizes coordinated redox, iron, lipid, and antioxidant defense pathways, whereas the senescence network focuses on inflammatory, cell-cycle, extracellular matrix, and hepatic stellate cell-associated processes. This network-based visualization aids in the discovery of hub genes, related pathway clusters, and potential regulators for further confirmation.

### 3.8 Ferroptosis–senescence cross-talk analysis identifies coordinated pro-ferroptotic and protective module interactions

To determine whether ferroptosis and senescence programs were functionally coordinated, FerroEnrich performed module-level cross-talk analysis between curated liver ferroptosis and liver senescence modules. Cross-talk was evaluated using three complementary outputs: a literature-based directional score matrix, combined cross talk strength matrix and a dataset-specific context.

The standard literature-based matrix defined the expected pro-ferroptotic and protective relationships between curated ferroptosis and senescence modules, establishing the biological framework for cross-talk interpretation in both the human NASH F4 and mouse NASH diet datasets (Fig. 5A; Fig. S4A, Supplementary Table S1, available at supplementary material  *Bioinformatics Advances* online). Cross-talk strength analysis revealed many high-ranking ferroptosis-senescence module combinations, demonstrating that ferroptosis-related lipid peroxidation, antioxidant defense, mitochondrial stress, and senescence/SASP programs are all regulated in tandem (Fig. 5B; Fig. S4B, Supplementary Table S1, available at supplementary material  *Bioinformatics Advances* online).

**Figure 5:**
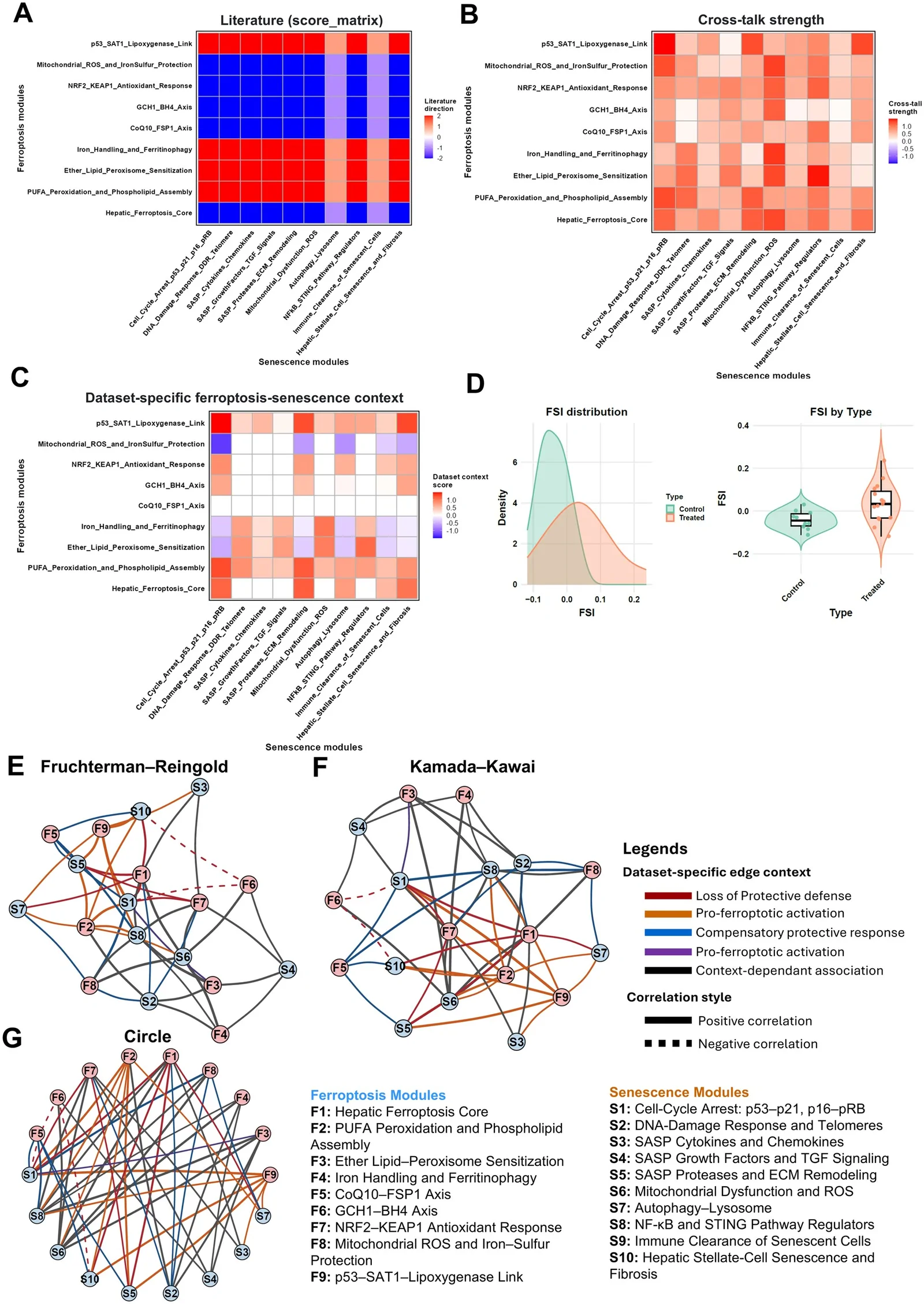
Ferroptosis–senescence cross-talk and Ferroptosis State Index (FSI) in the human NASH F4 dataset. (A) Literature-derived directional score matrix showing the curated biological relationships between ferroptosis and senescence modules. Positive scores indicate pro-ferroptotic module relationships, whereas negative scores indicate protective/anti-ferroptotic module relationships. (B) Cross-talk strength heatmap showing the magnitude of each ferroptosis–senescence module association. Cross-talk strength is used only to rank interaction magnitude and does not define biological direction. (C) Dataset-specific ferroptosis–senescence context heatmap integrating the curated ferroptosis-module role with the observed transcriptomic behavior in the mouse NASH diet dataset. (D) Distribution and violin/box plots of sample-level FSI values in healthy and NASH patient samples, illustrating differences in ferroptosis-associated transcriptomic activity between groups. (E–G) Network representations of ferroptosis–senescence module interactions using Fruchterman–Reingold, Kamada–Kawai, and circular layouts, respectively. Ferroptosis modules are denoted as F1–F9 and senescence modules as S1–S10. Edge colors represent dataset-specific biological context, including loss of protective defense, pro-ferroptotic activation, compensatory protective response, and context-dependent association, while solid and dashed edges indicate positive and negative correlations, respectively.

In the human NASH patient dataset, the dataset-specific context heatmap revealed increased pro-ferroptotic or loss-of-protection cross-talk across several module pairs, particularly involving p53-SAT1-lipoxygenase signaling, PUFA peroxidation and phospholipid assembly, hepatic ferroptosis core, and senescence modules related to cell-cycle arrest, mitochondrial dysfunction/ROS, immune clearance, and hepatic stellate-cell senescence/fibrosis (Fig. 5C; Table S3, Supplementary Table S1, available at supplementary material  *Bioinformatics Advances* online). In contrast, certain defensive modules, such as mitochondrial ROS/iron-sulfur protection and some antioxidant-response modules, exhibited weaker or compensatory context in specific senescence connections (Table S3, Supplementary Table S1, available at supplementary material  *Bioinformatics Advances* online). In accordance with these module-level trends, the ferroptosis-senescence index distribution shifted upward in NASH samples compared to controls, demonstrating enhanced ferroptosis-senescence coupling in human NASH (Fig. 5D).

In the mouse NASH diet dataset, cross-talk patterns were more broadly dominated by strong dataset-context scores, indicating higher pro-ferroptotic activation or loss of protective ferroptosis defence in association with senescence programs (Fig. S4C; Table S4, Supplementary Table S1, available at supplementary material  *Bioinformatics Advances* online). When these programs were diminished in the treated condition, prominent interactions included p53–SAT1–lipoxygenase signaling, PUFA peroxidation, ether lipid–peroxisome sensitization, iron handling/ferritinophagy, and protective defense modules like GCH1–BH4 and CoQ10–FSP1(Table S4, Supplementary Table S1, available at supplementary material  *Bioinformatics Advances* online). Following diet-induced liver damage, the FSI distribution similarly moved toward higher values in NASH diet-treated animals as compared to controls, indicating an intensified ferroptosis–senescence relationship (Fig. S4D, Supplementary Table S1, available at supplementary material  *Bioinformatics Advances* online).

Overall, both the human NASH F4 and mouse NASH diet datasets showed coordinated ferroptosis–senescence remodeling, according to the cross-talk analysis. Pro-ferroptotic activation or loss of protective ferroptosis-defense mechanisms associated with SASP, mitochondrial dysfunction, immunological remodeling, and fibrosis-related senescence predominated in these interactions, demonstrating conserved ferroptosis–senescence connection during NASH progression.

### 3.9 Ferroptosis Susceptibility Index (FSI)

FerroEnrich computed the Ferroptosis Susceptibility Index (FSI) to assess the balance of pro-ferroptotic and defensive ferroptosis module activity across samples. The FSI distribution in the human NASH F4 dataset showed a strong rightward shift when compared to healthy controls (Fig. 5D). Group-wise display revealed that NASH F4 samples exhibited greater FSI values than controls, indicating enhanced ferroptosis susceptibility and stronger activation of pro-ferroptotic module activity in comparison to protective ferroptosis-defense systems.

A similar trend was seen in the mouse NASH diet dataset. Compared to chow-fed controls, NASH diet samples exhibited a significant increase in FSI values, with the treated/NASH group having a right-shifted FSI distribution and higher group-level FSI scores (Fig. S4D, Supplementary Table S1, available at supplementary material  *Bioinformatics Advances* online). These findings indicate that ferroptosis susceptibility is consistently enhanced in both human advanced NASH and mouse diet-induced NASH models.

The FSI analysis confirms the occurrence of increased ferroptosis-associated vulnerability in NASH and shows that FerroEnrich can measure ferroptosis susceptibility at the sample level in both human and mouse transcriptome datasets.

### 3.10 Cross-talk network analysis

The top-ranked module-level correlations between hepatic ferroptosis and senescence programs were displayed by cross-talk network analysis. Ferroptosis modules in the human NASH F4 dataset shown significant linkage with modules associated to fibrosis, SASP, and senescence (Fig. 5E–G). The Fruchterman–Reingold, Circle, and Kamada–Kawai layouts all showed similar patterns, suggesting that the interactions were independent of the arrangement.

Notable associations encompassed PUFA peroxidation and phospholipid formation, p53–SAT1–lipoxygenase signaling pathways, iron handling/ferritinophagy, and ether lipid–peroxisome sensitization, which were correlated with cell-cycle inhibition, SASP proteases/ECM remodeling, SASP growth factor/TGF signaling, and hepatic stellate-cell senescence/fibrosis mechanisms. In the network, correlation direction is shown by edge style, dataset-specific biological context is indicated by edge color, and cross-talk intensity is represented by edge thickness; solid and dashed edges indicate positive and negative correlations, respectively.

The mouse NASH diet dataset revealed a similar network pattern (Fig. S4E–G, Supplementary Table S1, available at supplementary material  *Bioinformatics Advances* online), with substantial linkages to lipid peroxidation, iron handling, p53/SAT1-related ferroptosis, protective-defense modules, and senescence/fibrosis programs. Overall, these findings suggest the presence of ferroptosis-senescence cross-talk in human and mouse NASH, which is characterized by pro-ferroptotic activation or loss of protective ferroptosis-defense mechanisms linked to senescence remodeling.

## 4 Discussion

FerroEnrich was created as a useful, liver-focused web platform for analyzing ferroptosis activity in transcriptome datasets, notably in metabolic liver illness. Although ferroptosis is linked to NASH, fibrosis, cholestasis, cirrhosis, and hepatocellular cancer, converting ferroptosis gene lists into dataset-specific biological insights remains difficult. FerroEnrich bridges this gap by combining differential expression analysis, ferroptosis-prone and ferroptosis-resistant gene matching, Ferroptosis Index Value (FIV) calculation, liver-specific pathway enrichment, STRING-based network visualization, and ferroptosis-senescence cross-talk analysis into a single platform.

FerroEnrich complements existing ferroptosis resources, such as FerrDb and FERREG. These databases provide significant curated information on ferroptosis genes, regulators, markers, illness connections, and drug-related evidence, but they are not intended to serve as comprehensive transcriptome analytical platforms. FerroEnrich, on the other hand, allows users to upload human or mouse RNA-seq count matrices with metadata, compare groups using DESeq2, construct volcano plots, calculate dataset-level FIV scores, and assess liver-specific ferroptosis-senescence associations (Table 2). Thus, FerroEnrich does not replace FerrDb or FERREG, but rather integrates ferroptosis information into a dataset-driven analysis pipeline. In addition, FerroEnrich does not aim to replace established statistical or network-analysis tools such as DESeq2, clusterProfiler, STRINGdb, igraph, or visNetwork. Its novelty lies in the curated gene sets, FIV and FSI scoring systems, liver-specific module analysis, ferroptosis-senescence crosstalk interpretation, and association analysis within a single interactive platform. FerroEnrich provides dataset-specific behavior of liver-relevant prone and resistant programs, as opposed to assuming fixed disease or control directionality.

**Table 2:** Comparison of FerroEnrich with FerrDb and FERREG.

Feature	FerrDb	FERREG	FerroEnrich
**Primary purpose**	Curated ferroptosis gene and disease database	Ferroptosis regulator–target–drug–disease resource	Liver-focused ferroptosis/senescence transcriptomic analysis platform
**Ferroptosis drivers, suppressors, and markers**	Yes	Partial	Uses backend prone/resistant lists, but not a broad public annotation database
**Disease-associated ferroptosis gene annotations**	Yes	Yes	Limited to liver-focused interpretation and uploaded datasets
**Ferroptosis-related drug information**	Limited/available in database context	Strong drug/regulator information	Not currently included
**Regulator–target relationships**	Yes/limited	Yes	Not currently a major feature
**Literature-curated ferroptosis evidence**	Yes	Yes	Uses curated backend modules, but does not provide full literature browsing
**Query individual ferroptosis genes**	Yes	Yes	Limited; mainly analyzes genes present in user datasets
**Downloadable ferroptosis reference data**	Yes	Yes	Downloadable analysis outputs; backend resources can be shared with source code
**User RNA-seq count upload**	No	No	Yes
**Metadata-based group comparison**	No	No	Yes
**DESeq2 differential expression analysis**	No	No	Yes
**Volcano plot from user data**	No	No	Yes
**Dataset-specific prone/resistant gene matching**	No	No	Yes
**Ferroptosis Index Value**	No	No	Yes
**Liver-specific ferroptosis modules**	No	No	Yes
**Senescence module integration**	No	No	Yes
**GSEA/module enrichment from user data**	Not end-to-end	No	Yes
**STRING-based network from user data**	No	No	Yes
**Ferroptosis–senescence cross-talk analysis**	No	No	Yes
**Human and mouse demo datasets**	No	No	Yes
**Best use-case**	Annotating ferroptosis genes and checking known evidence	Exploring ferroptosis regulators, targets, drugs, and disease links	Interpreting ferroptosis/senescence activity in liver transcriptomic datasets

The table summarizes key differences among FerroEnrich, FerrDb, and FERREG, highlighting FerroEnrich as a liver-focused, dataset-driven platform for ferroptosis and senescence analysis.

The foundation model, Geneformer (Theodoris *et al*. 2023) is a strong representation-learning system that is trained on large-scale transcriptome datasets and may be used to model cellular states, gene-gene interactions, perturbation responses, and transcriptional representations. FerroEnrich supplements foundation models by offering a user-friendly, liver-focused bulk RNA-seq methodology that includes transparent ferroptosis scoring, curated module analysis, interaction networks, and interpretable outputs without requiring deep-learning experience. This strategy is especially beneficial in metabolism and hepatology research, where liver injury is characterized by coordinated lipid excess, oxidative stress, mitochondrial dysfunction, iron imbalance, inflammation, senescence, and fibrosis. Instead of focusing solely on individual ferroptosis genes, FerroEnrich divides ferroptosis pathway into functional modules such as PUFA peroxidation, iron management, GCH1/BH4 defense, CoQ10/FSP1 signaling, NRF2/KEAP1 antioxidant response, and mitochondrial ROS/iron-sulfur protection. When combined with the senescence and fibrosis modules, users can investigate ferroptosis as part of a larger metabolic damage network.

The inclusion of both human and mouse demo datasets promotes translational use. Users can test the approach with pre-existing datasets before applying the same analysis to their own count matrix and metadata files. FerroEnrich identified common ferroptosis-senescence remodeling patterns in human NASH F4 liver and mice NASH diet models, demonstrating its utility for cross-species interpretation.

FerroEnrich also has limits. Transcriptomic enrichment does not directly demonstrate ferroptotic cell death, and computational conclusions should be confirmed experimentally with lipid ROS assays, iron accumulation analysis, GPX4 protein assessment, 4-HNE or MDA measurement, or ferroptosis inhibitor rescue investigations. Furthermore, FIV should be understood in conjunction with module enrichment, matching prone/resistant gene tables, and biological context, rather than as a solitary diagnostic score. Future versions will include disease-specific modules, drug-response resources, support for single-cell and spatial transcriptomics, and enhanced automated report preparation.

Overall, FerroEnrich complements existing ferroptosis databases by providing a user-friendly, liver-focused, dataset-driven platform for ferroptosis and senescence interpretation. By connecting RNA-seq differential expression analysis with FIV scoring, pathway enrichment, network visualization, and cross-talk analysis, FerroEnrich provides a practical resource for metabolic liver disease research, biomarker prioritization, and hypothesis generation.

## Supplementary Material

vbag285_Supplementary_Data

## Data Availability

FerroEnrich is freely available at https://ferroenrich.shinyapps.io/ferroenrich/ The source code, curated gene sets, demo datasets and user manual are available at GitHub repository https://github.com/Munichandra23/FerroEnrich.git All datasets used for demonstration and validation are either included with the application or derived from publicly available transcriptomic resources cited in the manuscript.
