# Supplementary material for: FerroEnrich: an interactive web platform for liver-focused ferroptosis index scoring, enrichment, and ferroptosis–senescence cross-talk analysis
Source: Bioinform Adv. 2026 Sep 24;6(1):vbag285. doi: 10.1093/bioadv/vbag285 (PMC13652149; doi:10.1093/bioadv/vbag285)
Supplement: Supplementary file 1 [file vbag285_supplementary_data.zip › Supplementary_Tables.docx]

**Table S1. Liver-focused ferroptosis gene curation**. Genes were classified as ferroptosis-prone or ferroptosis-resistant based on FerroScore, biological rationale, liver-specific experimental evidence, assignment confidence, and supporting references.

| **Gene** | **Module** | **FerroScore** | **Biological Rational** | **Liver disease/model and experimental conditions** | **Liver-focused experimental evidence** | **Liver-evidence scope** | **Assignment confidence** | **Gene-level reference(s)** |
| --- | --- | --- | --- | --- | --- | --- | --- | --- |
| ACSL3 | Ferroptosis-prone | 17 | Promotes acyl-CoA activation of polyunsaturated fatty acids and incorporation into membrane phospholipids, increasing lipid peroxidation susceptibility. | human cells/tissue; ACSL4 is a predictive biomarker of sorafenib sensitivity in hepatocellular carcinoma. | Taken together, this study demonstrates that ACSL4 is essential for sorafenib-induced ferroptosis and useful for predicting sorafenib sensitivity in HCC. | Direct liver-focused experimental evidence | High; liver model with perturbation | Liver: PMID 32541921; FerrDb V2: PMID 36305834 |
| TP53 | Ferroptosis-prone | 9 | Stress-response regulator that can promote ferroptosis through metabolic, redox, and membrane transporter pathways in a context-dependent manner. | mouse; P53-dependent induction of ferroptosis is required for artemether to alleviate carbon tetrachloride-induced liver fibrosis and hepatic stellate cell activation. | P53-dependent induction of ferroptosis is required for artemether to alleviate carbon tetrachloride-induced liver fibrosis and hepatic stellate cell activation. | Direct liver-focused experimental evidence | High; liver model with perturbation | Liver: PMID 30321484; FerrDb V2: PMID 36305834 |
| HMOX1 | Ferroptosis-prone | 6 | Catalyzes heme degradation and can increase labile iron and oxidative stress, supporting iron-dependent lipid peroxidation. | human cells/tissue, mouse; Heme oxygenase 1 alleviates nonalcoholic steatohepatitis by suppressing hepatic ferroptosis. | Heme oxygenase 1 alleviates nonalcoholic steatohepatitis by suppressing hepatic ferroptosis. | Direct liver-focused experimental evidence | Moderate; context-dependent liver effect | Liver: PMID 37422643; FerrDb V2: PMID 36305834 |
| ALOX15 | Ferroptosis-prone | 5 | Lipoxygenase involved in enzymatic oxidation of polyunsaturated lipids, contributing to ferroptotic lipid peroxide accumulation. | rat; Lachnospiraceae-bacterium alleviates ischemia-reperfusion injury in steatotic donor liver by inhibiting ferroptosis via the Foxo3-Alox15 signaling pathway. | alleviates IRI in steatotic donor liver transplantation by inhibiting ferroptosis via the Foxo3-Alox15 axis, providing a potential therapeutic strategy to modulate gut microbiota to alleviate IRI following… | Direct liver-focused experimental evidence | Moderate; liver model | Liver: PMID 39882747; FerrDb V2: PMID 36305834 |
| IREB2 | Ferroptosis-prone | 5 | Regulates cellular iron metabolism and can increase iron-dependent ferroptosis susceptibility. | human cells/tissue, mouse; Iron regulatory protein 2 is required for artemether -mediated anti-hepatic fibrosis through ferroptosis pathway. | Iron regulatory protein 2 is required for artemether -mediated anti-hepatic fibrosis through ferroptosis pathway. | Direct liver-focused experimental evidence | High; liver model with perturbation | Liver: PMID 32947011; FerrDb V2: PMID 36305834 |
| NOX4 | Ferroptosis-prone | 5 | Generates reactive oxygen species and promotes oxidative stress that can enhance lipid peroxidation. | mouse; Caveolin-1 ameliorates hepatic injury in non-alcoholic fatty liver disease by inhibiting ferroptosis via the NOX4/ROS/GPX4 pathway. | Finally, NOX4 inhibitor (GLX351322) treatment increased CAV1 siRNA-mediated GPX4 expression and decreased the level of ROS-mediated ferroptosis. | Direct liver-focused experimental evidence | High; liver model with perturbation | Liver: PMID 39490677; FerrDb V2: PMID 36305834 |
| ALOX12 | Ferroptosis-prone | 4 | Lipoxygenase that contributes to oxidation of arachidonic acid-containing lipids and ferroptotic signaling. | mouse; TRPM2 Mediates Hepatic Ischemia-Reperfusion Injury via Ca2+-Induced Mitochondrial Lipid Peroxidation through Increasing ALOX12 Expression. | Collectively, these findings demonstrate a crucial role for TRPM2-mediated ferroptosis in hepatic IR injury via increased Ca2+-induced ALOX12 expression, indicating that pharmacological inhibition of TRPM2… | Direct liver-focused experimental evidence | High; liver model with perturbation | Liver: PMID 37275121; FerrDb V2: PMID 36305834 |
| TFRC | Ferroptosis-prone | 4 | Mediates transferrin-dependent iron uptake, increasing intracellular iron availability for Fenton chemistry and lipid oxidation. | human cells/tissue; Ceruloplasmin suppresses ferroptosis by regulating iron homeostasis in hepatocellular carcinoma cells. | Ceruloplasmin suppresses ferroptosis by regulating iron homeostasis in hepatocellular carcinoma cells. | Direct liver-focused experimental evidence | High; liver model with perturbation | Liver: PMID 32283255; FerrDb V2: PMID 36305834 |
| LPCAT3 | Ferroptosis-prone | 3 | Incorporates polyunsaturated fatty acids into phospholipids, increasing the lipid substrates required for ferroptosis. | mouse; Ginsenoside RK1 Promotes Hepatic Stellate Cell Glycolysis-Mediated Ferroptosis by Activating the HK2/ACSL4/LPCAT3/ALOX5 Signaling Pathway. | This study revealed that GRK1 targets HK2 in HSC-T6 cells to stimulate the ACSL4/LPCAT3/ALOX5 pathway, thereby inducing HSC ferroptosis and alleviating hepatic fibrosis. | Direct liver-focused experimental evidence | Moderate; liver model | Liver: PMID 40570179; FerrDb V2: PMID 36305834 |
| ATF3 | Ferroptosis-prone | 3 | Stress-inducible transcription factor linked to ferroptosis-related oxidative and inflammatory responses. | cell model; SEH1L siliencing induces ferroptosis and suppresses hepatocellular carcinoma progression via ATF3/HMOX1/GPX4 axis. | This study indicated that SEH1L siliencing could induce ferroptosis and suppresses hepatocellular carcinoma (HCC) progression via ATF3/HMOX1/GPX4 axis. | Direct liver-focused experimental evidence | High; liver model with perturbation | Liver: PMID 39095556; FerrDb V2: PMID 36305834 |
| SLC38A1 | Ferroptosis-prone | 3 | Amino-acid transporter associated with metabolic stress responses that may support ferroptosis-prone cellular states. | cell model; The Expression of Ferroptosis-Related Genes in Hepatocellular Carcinoma and Their Relationships With Prognosis. | The Expression of Ferroptosis-Related Genes in Hepatocellular Carcinoma and Their Relationships With Prognosis. | Liver-associated; limited direct regulatory evidence | Low-Moderate; liver association | Liver: PMID 40130081; FerrDb V2: PMID 36305834 |
| SIRT1 | Ferroptosis-prone | 3 | Stress and metabolism regulator with context-dependent roles in oxidative stress and ferroptosis-associated transcriptional programs. | mouse; Intestinal SIRT1 Deficiency Protects Mice from Ethanol-Induced Liver Injury by Mitigating Ferroptosis. | The protective effects of intestinal SIRT1 deficiency are mediated, at least partially, by mitigating hepatic ferroptosis. | Direct liver-focused experimental evidence | Moderate; context-dependent liver effect | Liver: PMID 31610175; FerrDb V2: PMID 36305834 |
| ACO1 | Ferroptosis-prone | 2 | Iron-responsive cytosolic aconitase involved in iron sensing and iron-metabolism regulation. | cell model; ENO1 suppresses cancer cell ferroptosis by degrading the mRNA of iron regulatory protein 1. | Moreover, through in vitro and in vivo experiments and clinical sample analysis, we identified IRP1 and Mfrn1 as tumor suppressors by inducing ferroptosis in HCC cells. | Direct liver-focused experimental evidence | High; liver model with perturbation | Liver: PMID 35121990; FerrDb V2: PMID 36305834 |
| ALOX5 | Ferroptosis-prone | 2 | Lipoxygenase involved in fatty-acid oxidation and lipid mediator production that can contribute to lipid peroxide generation. | mouse; Ginsenoside RK1 Promotes Hepatic Stellate Cell Glycolysis-Mediated Ferroptosis by Activating the HK2/ACSL4/LPCAT3/ALOX5 Signaling Pathway. | This study revealed that GRK1 targets HK2 in HSC-T6 cells to stimulate the ACSL4/LPCAT3/ALOX5 pathway, thereby inducing HSC ferroptosis and alleviating hepatic fibrosis. | Direct liver-focused experimental evidence | Moderate; liver model | Liver: PMID 40570179; FerrDb V2: PMID 36305834 |
| BACH1 | Ferroptosis-prone | 2 | Transcriptional regulator of heme and antioxidant pathways; increased activity can favor oxidative stress and ferroptosis sensitivity. | No direct liver-specific primary experiment identified; Mice; Mouse embryonic fibroblasts and mouse tissue-injury models | Promotes ferroptosis by repressing the transcription of a subset of the erastin-induced protective genes. | Cross-tissue only; no direct liver experiment identified | Low for liver-specific assignment | PMID: 31740582; FerrDb record: Driver, Validated; liver-focused evidence gap noted |
| EPAS1 | Ferroptosis-prone | 2 | Hypoxia-responsive transcription factor associated with redox remodeling and ferroptosis-related stress adaptation. | human cells/tissue, mouse; Screening of ferroptosis-related genes in sepsis-induced liver failure and analysis of immune correlation. | Screening of ferroptosis-related genes in sepsis-induced liver failure and analysis of immune correlation. | Liver-associated; limited direct regulatory evidence | Low-Moderate; liver association | Liver: PMID 35923893; FerrDb V2: PMID 36305834 |
| ELAVL1 | Ferroptosis-prone | 2 | RNA-binding protein reported to regulate stability of transcripts involved in ferroptosis and stress-response pathways. | human cells/tissue, mouse; Activation of ferritinophagy is required for the RNA-binding protein ELAVL1/HuR to regulate ferroptosis in hepatic stellate cells. | Attractively, ELAVL1 upregulation, ferritinophagy activation, and ferroptosis induction occurred in primary human HSCs from the collected human liver tissue. | Direct liver-focused experimental evidence | High; liver model with perturbation | Liver: PMID 30081711; FerrDb V2: PMID 36305834 |
| FTH1 | Ferroptosis-prone | 2 | Ferritin heavy chain involved in iron storage; included as prone in this database context because altered ferritin/iron handling can mark ferroptosis-associated iron remodeling. | human cells/tissue; H-ferritin suppression and pronounced mitochondrial respiration make Hepatocellular Carcinoma cells sensitive to RSL3-induced ferroptosis. | H-ferritin suppression and pronounced mitochondrial respiration make Hepatocellular Carcinoma cells sensitive to RSL3-induced ferroptosis. | Direct liver-focused experimental evidence | Moderate; context-dependent liver effect | Liver: PMID 33892112; FerrDb V2: PMID 36305834 |
| FTL | Ferroptosis-prone | 2 | Ferritin light chain involved in iron storage; altered expression reflects ferroptosis-associated iron metabolism remodeling. | human cells/tissue; Integrated Analysis Reveals Critical Ferroptosis Regulators and FTL Contribute to Cancer Progression in Hepatocellular Carcinoma. | Further knockdown of FTL was determined to significantly inhibit cell proliferation and migration in HCC. | Liver-associated; limited direct regulatory evidence | Low-Moderate; liver association | Liver: PMID 35651950; FerrDb V2: PMID 36305834 |
| IL6 | Ferroptosis-prone | 2 | Inflammatory cytokine linked to oxidative stress, iron handling, and ferroptosis-associated inflammatory signaling. | human cells/tissue, mouse; IL6 Derived from Macrophages under Intermittent Hypoxia Exacerbates NAFLD by Promoting Ferroptosis via MARCH3-Led Ubiquitylation of GPX4. | The increased secretion of IL6 by macrophages can promote the expression of MARCH3 in hepatocytes under intermittent conditions, and subsequently promote the ubiquitination and degradation of GPX4 to regulate | Direct liver-focused experimental evidence | Moderate; context-dependent liver effect | Liver: PMID 39229924; FerrDb V2: PMID 36305834 |
| MAPK3 | Ferroptosis-prone | 2 | ERK/MAPK signaling component involved in stress-response pathways that can regulate ferroptosis-associated cellular programs. | rat; Activity-Dependent Effects of ERK1/2 on Hepatic Ischemia-Reperfusion Injury. | Our data demonstrate that ERK1/2 activity is required for hepatic cells to tolerate IRI. | Direct liver-focused experimental evidence | Moderate; liver model | Liver: PMID 40796392; FerrDb V2: PMID 36305834 |
| MAPK1 | Ferroptosis-prone | 2 | ERK/MAPK signaling component involved in oxidative stress signaling and ferroptosis-related pathway regulation. | rat; Activity-Dependent Effects of ERK1/2 on Hepatic Ischemia-Reperfusion Injury. | Our data demonstrate that ERK1/2 activity is required for hepatic cells to tolerate IRI. | Direct liver-focused experimental evidence | Moderate; liver model | Liver: PMID 40796392; FerrDb V2: PMID 36305834 |
| MAPK8 | Ferroptosis-prone | 2 | JNK/MAPK stress kinase associated with oxidative stress, cell injury, and ferroptosis-related signaling. | cell model; Phosphorylated FOXQ1, a novel substrate of JNK1, inhibits sorafenib-induced ferroptosis by activating ETHE1 in hepatocellular carcinoma. | Collectively, our study implicated the JNK1-FOXQ1-ETHE1 axis in HCC ferroptosis induced by sorafenib, providing mechanistic insight into sensitivity to sorafenib therapy of HCC. | Direct liver-focused experimental evidence | Moderate; liver model | Liver: PMID 38839744; FerrDb V2: PMID 36305834 |
| TF | Ferroptosis-prone | 2 | Transferrin supports systemic and cellular iron transport, increasing the iron supply required for iron-dependent lipid peroxidation. | human cells/tissue, mouse; Hepatic transferrin plays a role in systemic iron homeostasis and liver ferroptosis. | Hepatic transferrin plays a role in systemic iron homeostasis and liver ferroptosis. | Direct liver-focused experimental evidence | Moderate; context-dependent liver effect | Liver: PMID 32374849; FerrDb V2: PMID 36305834 |
| FAR1 | Ferroptosis-prone | 2 | Fatty acyl-CoA reductase linked to ether lipid/plasmalogen metabolism, influencing lipid composition and ferroptosis sensitivity. | rat; Maternal protein deficiency impairs peroxisome biogenesis and leads to oxidative stress and ferroptosis in liver of fetal growth restriction offspring. | Double knock down (DKD) of PEX14 and fatty acyl-CoA reductase 1 (FAR1) revealed that PEX14 KD-induced ferroptosis was related with enhanced FAR1 level. | Direct liver-focused experimental evidence | High; liver model with perturbation | Liver: PMID 37657642; FerrDb V2: PMID 36305834 |
| BAP1 | Ferroptosis-prone | 2 | Tumor suppressor reported to promote ferroptosis through regulation of cystine transporter and chromatin-associated stress pathways. | human cells/tissue; Somatic mutation and expression of BAP1 in hepatocellular carcinoma: an indicator for ferroptosis and immune checkpoint inhibitor therapies. | BAP1 is a promising prognostic marker for survival of HCC and may act as a complementary indicator for patients to receive ferroptosis-promoting therapy or immunotherapy. | Liver-associated; limited direct regulatory evidence | Low-Moderate; liver association | Liver: PMID 34976173; FerrDb V2: PMID 36305834 |
| HMGB1 | Ferroptosis-prone | 2 | Damage-associated molecular pattern linked to inflammatory stress and ferroptosis-associated cell injury signaling. | mouse; Involvement of Ferroptosis in Diabetes-Induced Liver Pathology. | This improvement was due to the Fer-1-induced attenuation of ferroptotic events in the liver of diabetic mice, such as accumulation of pro-oxidative parameters (iron, lipofuscin, 4-HNE), decrease in… | Liver-associated; limited direct regulatory evidence | Low-Moderate; liver association | Liver: PMID 36012572; FerrDb V2: PMID 36305834 |
| NR1D1 | Ferroptosis-prone | 2 | Nuclear receptor involved in metabolic and redox regulation, with reported links to ferroptosis-associated transcriptional control. | mouse; Liquiritigenin alleviates liver fibrosis through the nuclear receptor subfamily 1 group D member 1-regulated ferroptosis signaling pathway. | These findings suggest that LQ can inhibit ferroptosis by modulating the NR1D1-SLC7A11 signaling pathway in liver fibrosis. | Direct liver-focused experimental evidence | High; liver model with perturbation | Liver: PMID 42068875; FerrDb V2: PMID 36305834 |
| SLC11A2 | Ferroptosis-prone | 2 | Divalent metal transporter involved in iron uptake and intracellular iron accumulation, supporting ferroptosis susceptibility. | human cells/tissue; Decursin induces ferroptosis via the NRF2/GPX4/SLC11A2 axis and suppresses migration in hepatocellular carcinoma. | Decursin induces ferroptosis via the NRF2/GPX4/SLC11A2 axis and suppresses migration in hepatocellular carcinoma. | Direct liver-focused experimental evidence | High; liver model with perturbation | Liver: PMID 41496356; FerrDb V2: PMID 36305834 |
| SLC1A5 | Ferroptosis-prone | 2 | Amino-acid transporter associated with glutamine metabolism and ferroptosis-relevant metabolic remodeling. | mouse; Tamoxifen regulates ferroptosis of hepatocytes by targeting SLC1A5 to activate hepatic stellate cells and liver fibrosis. | Our study suggests that TAM induces hepatocyte ferroptosis through SLC1A5, leading to HSC activation and liver fibrosis. | Direct liver-focused experimental evidence | High; liver model with perturbation | Liver: PMID 40436235; FerrDb V2: PMID 36305834 |
| SIRT3 | Ferroptosis-prone | 2 | Mitochondrial stress regulator with context-dependent effects on ROS generation and ferroptosis-associated mitochondrial metabolism. | cell model; Forsythiaside A ameliorates acetaminophen-induced liver injury by suppressing ferroptosis through activating USP11/SIRT3-mediated mitophagy. | FTA ameliorates AILI by suppressing ferroptosis through activating USP11/SIRT3-mediated mitophagy, revealing a novel mechanistic pathway. | Direct liver-focused experimental evidence | Moderate; context-dependent liver effect | Liver: PMID 41338315; FerrDb V2: PMID 36305834 |
| STING1 | Ferroptosis-prone | 2 | Innate immune signaling adaptor associated with inflammatory stress responses and ferroptosis-linked tissue injury. | No direct liver-specific primary experiment identified; Human; Human pancreatic-cancer cells; mitochondrial-fusion mechanism | STING1 Promotes Ferroptosis Through MFN1/2-Dependent Mitochondrial Fusion. | Cross-tissue only; no direct liver experiment identified | Low for liver-specific assignment | PMID: 34195205; FerrDb record: Driver, Validated; liver-focused evidence gap noted |
| GPX4 | Ferroptosis-resistant | 16 | Central lipid peroxide detoxification enzyme that suppresses ferroptosis by reducing phospholipid hydroperoxides. | mouse; Decreased autophagosome biogenesis, reduced NRF2, and enhanced ferroptotic cell death are underlying molecular mechanisms of non-alcoholic fatty liver disease. | Decreased autophagosome biogenesis, reduced NRF2, and enhanced ferroptotic cell death are underlying molecular mechanisms of non-alcoholic fatty liver disease. | Direct liver-focused experimental evidence | High; liver model with perturbation | Liver: PMID 36495698; FerrDb V2: PMID 36305834 |
| NFE2L2 | Ferroptosis-resistant | 16 | Master antioxidant transcription factor that induces cytoprotective, glutathione, detoxification, and iron-handling programs. | human cells/tissue, mouse; Nrf2-Mediated Ferroptosis Inhibition Exerts a Protective Effect on Acute-on-Chronic Liver Failure. | Collectively, Nrf2 plays a protective role in ACLF progression through repressing ferroptosis, which provides promising therapeutic cues for ACLF. | Direct liver-focused experimental evidence | Moderate; context-dependent liver effect | Liver: PMID 35480867; FerrDb V2: PMID 36305834 |
| SLC7A11 | Ferroptosis-resistant | 14 | Cystine/glutamate antiporter subunit that supports cystine uptake, glutathione synthesis, and GPX4-dependent ferroptosis resistance. | mouse; Inhibiting xCT/SLC7A11 induces ferroptosis of myofibroblastic hepatic stellate cells but exacerbates chronic liver injury. | Inhibiting xCT/SLC7A11 induces ferroptosis of myofibroblastic hepatic stellate cells but exacerbates chronic liver injury. | Direct liver-focused experimental evidence | Moderate; context-dependent liver effect | Liver: PMID 33991158; FerrDb V2: PMID 36305834 |
| SCD | Ferroptosis-resistant | 5 | Stearoyl-CoA desaturase increases monounsaturated fatty acids, reducing membrane PUFA peroxidation and ferroptosis sensitivity. | human cells/tissue; URI alleviates tyrosine kinase inhibitors-induced ferroptosis by reprogramming lipid metabolism in p53 wild-type liver cancers. | The combination of SCD1 inhibitor aramchol and deuterated sorafenib derivative donafenib displays promising anti-tumor effects in p53-wild type HCC patient-derived organoids and xenografted tumors. | Direct liver-focused experimental evidence | High; liver model with perturbation | Liver: PMID 37805657; FerrDb V2: PMID 36305834 |
| CBS | Ferroptosis-resistant | 4 | Transsulfuration-pathway enzyme supporting cysteine and glutathione availability for anti-ferroptotic defense. | mouse; A pharmacological probe identifies cystathionine β-synthase as a new negative regulator for ferroptosis. | Importantly, inhibition of CBS triggers ferroptosis in hepatocellular carcinoma. | Direct liver-focused experimental evidence | High; liver model with perturbation | Liver: PMID 30258181; FerrDb V2: PMID 36305834 |
| FTMT | Ferroptosis-resistant | 4 | Mitochondrial ferritin that sequesters iron and limits mitochondrial iron-driven oxidative injury. | No direct liver-specific primary experiment identified; Mice, fly; Mouse and Drosophila neuronal models | Overexpression significantly inhibited erastin-induced ferroptosis. | Cross-tissue only; no direct liver experiment identified | Low for liver-specific assignment | PMID: 28066232; FerrDb record: Suppressor, Validated; liver-focused evidence gap noted |
| MTOR | Ferroptosis-resistant | 4 | Nutrient-sensing kinase that regulates growth, metabolism, autophagy, and stress responses linked to ferroptosis resistance. | mouse; Malic Enzyme 1 as a Novel Anti-Ferroptotic Regulator in Hepatic Ischemia/Reperfusion Injury. | Finally, PTEN inhibitor, mTOR activator, or SREBP1 over-expression all increase hepatic NADPH, block ferroptosis, and protect liver against I/R injury. | Direct liver-focused experimental evidence | High; liver model with perturbation | Liver: PMID 36840630; FerrDb V2: PMID 36305834 |
| AIFM2 | Ferroptosis-resistant | 3 | Also known as FSP1; suppresses ferroptosis through CoQ10-dependent lipid radical trapping at membranes. | human cells/tissue; Ferroptosis Suppressor Protein 1 Inhibition Promotes Tumor Ferroptosis and Anti-tumor Immune Responses in Liver Cancer. | FSP1 inhibition therefore represents a new therapeutic strategy for HCC. | Direct liver-focused experimental evidence | Moderate; liver model | Liver: PMID 36893885; FerrDb V2: PMID 36305834 |
| CDN1 | Ferroptosis-resistant | 3 | Cell-cycle/stress-response gene in the backend database; associated with protective stress adaptation and ferroptosis resistance annotation. | human cells/tissue, mouse; CDKN1A and EGR1 are key genes for endoplasmic reticulum stress-induced ferroptosis in MASH. | In summary, all results indicate that CDKN1A and EGR1 are key genes driving ER stress-induced ferroptosis in MASH. | Direct liver-focused experimental evidence | High; liver model with perturbation | Liver: PMID 40414463; FerrDb V2: PMID 36305834 |
| SLC40A1 | Ferroptosis-resistant | 3 | Ferroportin iron exporter that lowers intracellular iron accumulation and can reduce iron-dependent ferroptosis pressure. | mouse, bovine; Quercetin alleviates LPS/iE-DAP-induced liver injury by suppressing ferroptosis via regulating ferritinophagy and intracellular iron efflux. | Based on these findings, we concluded that LPS/iE-DAP-induced liver injury by triggering ferroptosis through regulating IL-6/STAT3/ferritinophagy-dependent iron release and… | Direct liver-focused experimental evidence | High; liver model with perturbation | Liver: PMID 39986118; FerrDb V2: PMID 36305834 |
| SATS3 | Ferroptosis-resistant | 3 | STAT3-related signaling annotation in the backend database; associated with survival and stress-response programs that can oppose ferroptosis. | human cells/tissue, mouse; SERPINE2-mediated activation of JAK2/STAT3 facilitates NRF2 nuclear translocation and GCLC transcription to confer ferroptosis resistance and lenvatinib resistance… | This study established the SERPINE2-JAK2/STAT3-NRF2-GCLC signaling axis as a key mechanism driving ferroptosis resistance and lenvatinib treatment failure. | Direct liver-focused experimental evidence | Moderate; context-dependent liver effect | Liver: PMID 42251352; FerrDb V2: PMID 36305834 |
| FXN | Ferroptosis-resistant | 3 | Frataxin regulates mitochondrial iron-sulfur cluster biology and protects against mitochondrial iron dysregulation. | human cells/tissue, mouse; Oxidative stress-dependent frataxin inhibition mediated alcoholic hepatocytotoxicity through ferroptosis. | Oxidative stress-dependent frataxin inhibition mediated alcoholic hepatocytotoxicity through ferroptosis. | Direct liver-focused experimental evidence | High; liver model with perturbation | Liver: PMID 33017621; FerrDb V2: PMID 36305834 |
| HSF1 | Ferroptosis-resistant | 3 | Heat-shock transcription factor that activates proteostasis and stress-protective programs linked to ferroptosis resistance. | mouse; Gandouling ameliorates liver injury in Wilson's disease through the inhibition of ferroptosis by regulating the HSF1/HSPB1 pathway. | In conclusion, GDL shows promise in alleviating liver injury in WD by inhibiting ferroptosis through modulation of the HSF1/HSPB1 pathway, suggesting its potential as a novel therapeutic agent for treating | Direct liver-focused experimental evidence | Moderate; liver model | Liver: PMID 39223962; FerrDb V2: PMID 36305834 |
| HSPA5 | Ferroptosis-resistant | 3 | ER chaperone GRP78/BiP that supports unfolded protein response and can protect cells from ferroptosis-associated stress. | mouse; Targeting GCS regulates the NRF2 axis to inhibit hepatocyte endoplasmic reticulum stress-ferroptosis improving immune-mediated liver injury. | Targeting GCS regulates the NRF2 axis to inhibit hepatocyte endoplasmic reticulum stress-ferroptosis improving immune-mediated liver injury. | Direct liver-focused experimental evidence | High; liver model with perturbation | Liver: PMID 40946670; FerrDb V2: PMID 36305834 |
| LCN2 | Ferroptosis-resistant | 3 | Iron-binding lipocalin involved in iron trafficking; contextually annotated with ferroptosis-protective iron-handling responses. | human cells/tissue, mouse; A targetable LIFR-NF-κB-LCN2 axis controls liver tumorigenesis and vulnerability to ferroptosis. | Notably, an LCN2-neutralizing antibody enhances the ferroptosis-inducing and anticancer effects of sorafenib on HCC patient-derived xenograft tumors with low LIFR expression and high LCN2 expression. | Direct liver-focused experimental evidence | High; liver model with perturbation | Liver: PMID 34921145; FerrDb V2: PMID 36305834 |
| AKR1C1 | Ferroptosis-resistant | 2 | Aldo-keto reductase involved in detoxification of lipid peroxidation-derived aldehydes, limiting oxidative lipid damage. | human cells/tissue; Zingiberensis new saponin reverses sorafenib resistance by targeting lncRNA TCONS-00026762/AKR1C1 and modulating autophagy and ferroptosis in hepatocellular… | Our findings suggest that ZnS inhibits autophagy, promotes ferroptosis, and enhances sensitivity to sorafenib in HCC cells through the TCONS-00026762/AKR1C1 axis. | Direct liver-focused experimental evidence | High; liver model with perturbation | Liver: PMID 41319876; FerrDb V2: PMID 36305834 |
| MLST8 | Ferroptosis-resistant | 2 | mTOR complex component involved in nutrient and stress signaling, supporting cell survival pathways. | No direct liver-specific primary experiment identified; Mice; Mouse memory CD4+ T cells; mTORC1 perturbation | Collectively, these results indicate that mTORC1 attenuates ferroptosis by maintaining intracellular GPX4 activity and blocking lipid peroxidation. | Cross-tissue only; no direct liver experiment identified | Low for liver-specific assignment | PMID: 36722887; FerrDb record: Suppressor, Validated; liver-focused evidence gap noted |
| ATF4 | Ferroptosis-resistant | 2 | Integrated stress-response transcription factor that can promote amino-acid, cysteine, and glutathione defense programs. | human cells/tissue, mouse; ATF4 suppresses hepatocarcinogenesis by inducing SLC7A11 (xCT) to block stress-related ferroptosis. | Although ATF4 ablation prevents hepatic steatosis, it also increases susceptibility to ferroptosis, due to decreased expression of the cystine/glutamate antiporter SLC7A11. | Direct liver-focused experimental evidence | Moderate; context-dependent liver effect | Liver: PMID 36996941; FerrDb V2: PMID 36305834 |
| HIF1A | Ferroptosis-resistant | 2 | Hypoxia-responsive factor that regulates metabolic adaptation and can support ferroptosis-protective remodeling in some contexts. | No direct liver-specific primary experiment identified; Human, mice; Human hepatocellular-carcinoma cells under hypoxia (context-specific) | Destabilizing HIF1A facilitated ferroptotic tumor cell death. | Cross-tissue only; no direct liver experiment identified | Low for liver-specific assignment | PMID: 35304827; FerrDb record: Suppressor, Validated; liver-focused evidence gap noted |
| GCLC | Ferroptosis-resistant | 2 | Catalytic subunit of glutamate-cysteine ligase required for glutathione synthesis and antioxidant defense. | mouse; Ethyl carbamate triggers ferroptosis in liver through inhibiting GSH synthesis and suppressing Nrf2 activation. | EC-triggered GSH depletion mainly depended on suppressed GSH synthesis via inhibition of SLC7A11 and GCLC expressions. | Direct liver-focused experimental evidence | Moderate; liver model | Liver: PMID 35623314; FerrDb V2: PMID 36305834 |
| NF2 | Ferroptosis-resistant | 2 | Regulator of Hippo/stress signaling with reported ferroptosis-modulating activity in curated ferroptosis resources. | No direct liver-specific primary experiment identified; Human, mice; Human mesothelioma/cancer cells and mouse xenografts | Genetic inactivation of NF2 rendered cancer cells more sensitive to ferroptosis. Mediates cell density-dependent inhibition of ferroptosis. | Cross-tissue only; no direct liver experiment identified | Low for liver-specific assignment | PMID: 31341276; FerrDb record: Suppressor, Validated; liver-focused evidence gap noted |
| PROM2 | Ferroptosis-resistant | 2 | Promotes ferritin-containing multivesicular body/exosome release and reduces intracellular iron burden. | No direct liver-specific primary experiment identified; Human; Human breast-cancer cells and ferroptotic-stress models | Induced by ferroptotic stress and promotes resistance to ferroptotic cell death. Facilitates ferroptosis resistance in mammary epithelial and breast carcinoma cells. | Cross-tissue only; no direct liver experiment identified | Low for liver-specific assignment | PMID: 31735663; FerrDb record: Suppressor, Validated; liver-focused evidence gap noted |
| RPTOR | Ferroptosis-resistant | 2 | mTORC1 scaffold component involved in nutrient signaling and stress adaptation relevant to ferroptosis resistance. | No direct liver-specific primary experiment identified; Mice; Mouse cells and tissue models; mTORC1 perturbation | Collectively, these results indicate that mTORC1 attenuates ferroptosis by maintaining intracellular GPX4 activity and blocking lipid peroxidation. | Cross-tissue only; no direct liver experiment identified | Low for liver-specific assignment | PMID: 36722887; FerrDb record: Suppressor, Validated; liver-focused evidence gap noted |
| GCH1 | Ferroptosis-resistant | 2 | Generates tetrahydrobiopterin-dependent antioxidant capacity and lipid remodeling that protects against ferroptosis. | mouse; Cordycepin Alleviates Non Alcoholic Fatty Liver Disease Progression by Stabilizing GCH1 to Inhibit Ferroptosis. | COR elevates GCH1 levels to inhibit ferroptosis. | Direct liver-focused experimental evidence | High; liver model with perturbation | Liver: PMID 42560344; FerrDb V2: PMID 36305834 |
| HAMP | Ferroptosis-resistant | 2 | Hepcidin regulates systemic iron homeostasis and limits iron export/redistribution, influencing ferroptosis-related iron balance. | mouse; ACSS2 protects against alcohol-induced hepatocyte ferroptosis through regulation of hepcidin expression. | ACSS2 deficiency downregulates HAMP1/2, causing systemic iron dyshomeostasis and ferroptosis, which is restored by overexpression of HAMP1/2. | Direct liver-focused experimental evidence | High; liver model with perturbation | Liver: PMID 40593779; FerrDb V2: PMID 36305834 |

**Table S2. Gene set enrichment analysis of liver ferroptosis and senescence modules in the mouse NASH diet dataset.** This table summarizes enrichment results from NASH diet-treated and chow diet-treated mouse, with n = 5 per group, including module description, gene set size, enrichment score, normalized enrichment score, p-value, adjusted p-value, rank, core enrichment genes, and dataset category.

| **Description** | **setSize** | **Enrichment**  **Score** | **NES** | **pvalue** | **padj** | **rank** | **core_enrichment** | **Dataset** |
| --- | --- | --- | --- | --- | --- | --- | --- | --- |
| GCH1_BH4_Axis | 5 | -0.88 | -1.80 | 0.0027 | 0.02463 | 72 | GCH1/QDPR | Liver_ferroptosis |
| Hepatic_Ferroptosis_Core | 18 | -0.58 | -1.73 | 0.0107 | 0.048349 | 2872 | MDH1/GPX1/ME1/SLC3A2/GCLC/SLC38A2/GCLM/GLS2/  TXNRD1/IDH1/SLC25A1 | Liver_ferroptosis |
| NRF2_KEAP1_Antioxidant_Response | 18 | -0.55 | -1.65 | 0.0177 | 0.053382 | 2362 | KEAP1/GLRX2/SOD1/SOD2/GCLC/GCLM/TXNRD1/PRDX1 | Liver_ferroptosis |
| PUFA_Peroxidation_and_Phospholipid  _Assembly | 19 | 0.50 | 1.40 | 0.0779 | 0.175477 | 1497 | ELOVL5/LPCAT2/ACSL4/FADS2/PLA2G4A | Liver_ferroptosis |
| Iron_Handling_and_Ferritinophagy | 15 | 0.47 | 1.24 | 0.2126 | 0.38277 | 1612 | FTH1/HMOX1/SLC39A14/CP | Liver_ferroptosis |
| Mitochondrial_ROS_and_IronSulfur_Protection | 16 | -0.39 | -1.12 | 0.3294 | 0.49415 | 5698 | NDUFS1/SDHB/FDX1/CISD1/UQCRC2/DLST/NNT/NDUFA9/SOD2/  NDUFS2 | Liver_ferroptosis |
| p53_SAT1_Lipoxygenase_Link | 8 | 0.43 | 0.95 | 0.5304 | 0.59681 | 3409 | CDKN1A/SESN2/SAT1/ALOX15 | Liver_ferroptosis |
| Ether_Lipid_Peroxisome_Sensitization | 17 | 0.35 | 0.95 | 0.5292 | 0.59681 | 727 | PLA2G6/FAR1/PEX11A | Liver_ferroptosis |
| CoQ10_FSP1_Axis | 14 | -0.25 | -0.69 | 0.8735 | 0.87358 | 1251 | COQ10A/COQ5/COQ8B/COQ8A | Liver_ferroptosis |
| Hepatic_Stellate_Cell_Senescence_and  _Fibrosis | 15 | 0.85 | 2.29 | 4.39E-07 | 4.39E-06 | 1217 | COL1A1/COL3A1/COL1A2/LOXL2/LAMB1/PDGFRB/ACTA2/LAMA2/LOX | Liver_senescence |
| SASP_Proteases_ECM_Remodeling | 10 | 0.91 | 2.17 | 9.21E-07 | 4.60E-06 | 761 | MMP12/COL1A1/COL3A1/PLAU/TIMP2/TIMP1 | Liver_senescence |
| Immune_Clearance_of_Senescent_Cells | 8 | 0.75 | 1.66 | 0.0131 | 0.04390 | 3391 | VCAM1/KLRK1/CX3CL1/ULBP1/ICAM1/GZMB/PRF1 | Liver_senescence |
| SASP_GrowthFactors_TGF_Signals | 11 | 0.69 | 1.70 | 0.0232 | 0.05808 | 754 | IGFBP7/IGFBP3/TGFB1/FGF2 | Liver_senescence |
| SASP_Cytokines_Chemokines | 10 | 0.68 | 1.61 | 0.0314 | 0.0628 | 561 | CCL2/CCL5/CXCL2/CXCL10 | Liver_senescence |
| Mitochondrial_Dysfunction_ROS | 10 | -0.62 | -1.54 | 0.0397 | 0.06633 | 4475 | NDUFS1/PRDX5/UQCRC2/NDUFA9/GPX1/TFAM/SIRT3/SOD2/  PPARGC1A | Liver_senescence |
| Cell_Cycle_Arrest_p53_p21_p16_pRB | 12 | 0.60 | 1.51 | 0.0575 | 0.08222 | 3205 | CDKN1A/CCNA2/CCNE1/MDM4/CCND1/CDKN2A | Liver_senescence |
| Autophagy_Lysosome | 14 | -0.52 | -1.44 | 0.0837 | 0.10463 | 2991 | CTSL/ATG5/ATG12/ATG3/SQSTM1/LAMP2 | Liver_senescence |
| NFkB_STING_Pathway_Regulators | 10 | -0.35 | -0.87 | 0.6244 | 0.69381 | 2630 | TBK1/IKBKB/RELA/MAP3K7/TAB3/TAB2 | Liver_senescence |
| DNA_Damage_Response_DDR_Telomere | 11 | 0.20 | 0.50 | 0.9802 | 0.98025 | 4628 | CHEK2/CHEK1/RTEL1/TERF2 | Liver_senescence |

**Table S3. Ferroptosis–senescence cross-talk in the human NASH dataset.** Cross-talk between curated liver ferroptosis and senescence modules in human NASH patients, including module role, literature direction, correlation, cross-talk strength, module-level expression changes, and dataset-specific interpretation.

| **Ferroptosis Modules** | **Senescence Modules** | **Curated module role** | **Literature direction** | **Correlation** | **CrossTalk Strength** | **Ferro mean log2FC** | **Ferro_direction** | **Senescence mean log2FC** | **Senescence direction** | **Dataset context category** | **Dataset specific interpretation** |
| --- | --- | --- | --- | --- | --- | --- | --- | --- | --- | --- | --- |
| p53_SAT1_Lipoxygenase_Link | Cell_Cycle_Arrest_p53_p21_p16_pRB | Prone / pro-ferroptotic | 2 | 0.922 | 1.843 | 0.17 | Up | 0.374 | Up | Pro-ferroptotic activation | Pro-ferroptotic module increased with senescence/SASP activation |
| p53_SAT1_Lipoxygenase_Link | Hepatic_Stellate_Cell_Senescence_and_Fibrosis | Prone / pro-ferroptotic | 2 | 0.92 | 1.84 | 0.17 | Up | 0.537 | Up | Pro-ferroptotic activation | Pro-ferroptotic module increased with senescence/SASP activation |
| p53_SAT1_Lipoxygenase_Link | SASP_Proteases_ECM_Remodeling | Prone / pro-ferroptotic | 2 | 0.918 | 1.836 | 0.17 | Up | 0.614 | Up | Pro-ferroptotic activation | Pro-ferroptotic module increased with senescence/SASP activation |
| PUFA_Peroxidation_and_Phospholipid_Assembly | Hepatic_Stellate_Cell_Senescence_and_Fibrosis | Prone / pro-ferroptotic | 2 | 0.905 | 1.811 | 0.219 | Up | 0.537 | Up | Pro-ferroptotic activation | Pro-ferroptotic module increased with senescence/SASP activation |
| p53_SAT1_Lipoxygenase_Link | SASP_GrowthFactors_TGF_Signals | Prone / pro-ferroptotic | 2 | 0.898 | 1.796 | 0.17 | Up | -0.671 | Down | Pro-ferroptotic activation | Pro-ferroptotic module increased while senescence program is reduced |
| Ether_Lipid_Peroxisome_Sensitization | Hepatic_Stellate_Cell_Senescence_and_Fibrosis | Prone / pro-ferroptotic | 2 | 0.892 | 1.784 | -0.155 | Down | 0.537 | Up | Reduced pro-ferroptotic program | Reduced pro-ferroptotic module despite senescence/SASP activation |
| PUFA_Peroxidation_and_Phospholipid_Assembly | SASP_GrowthFactors_TGF_Signals | Prone / pro-ferroptotic | 2 | 0.878 | 1.756 | 0.219 | Up | -0.671 | Down | Pro-ferroptotic activation | Pro-ferroptotic module increased while senescence program is reduced |
| PUFA_Peroxidation_and_Phospholipid_Assembly | SASP_Proteases_ECM_Remodeling | Prone / pro-ferroptotic | 2 | 0.874 | 1.748 | 0.219 | Up | 0.614 | Up | Pro-ferroptotic activation | Pro-ferroptotic module increased with senescence/SASP activation |
| Ether_Lipid_Peroxisome_Sensitization | SASP_GrowthFactors_TGF_Signals | Prone / pro-ferroptotic | 2 | 0.868 | 1.735 | -0.155 | Down | -0.671 | Down | Context-dependent association | Pro-ferroptotic module reduced with senescence program suppression |
| Ether_Lipid_Peroxisome_Sensitization | SASP_Proteases_ECM_Remodeling | Prone / pro-ferroptotic | 2 | 0.824 | 1.649 | -0.155 | Down | 0.614 | Up | Reduced pro-ferroptotic program | Reduced pro-ferroptotic module despite senescence/SASP activation |
| p53_SAT1_Lipoxygenase_Link | SASP_Cytokines_Chemokines | Prone / pro-ferroptotic | 2 | 0.815 | 1.631 | 0.17 | Up | -0.239 | Down | Pro-ferroptotic activation | Pro-ferroptotic module increased while senescence program is reduced |
| PUFA_Peroxidation_and_Phospholipid_Assembly | Cell_Cycle_Arrest_p53_p21_p16_pRB | Prone / pro-ferroptotic | 2 | 0.815 | 1.629 | 0.219 | Up | 0.374 | Up | Pro-ferroptotic activation | Pro-ferroptotic module increased with senescence/SASP activation |
| Ether_Lipid_Peroxisome_Sensitization | Cell_Cycle_Arrest_p53_p21_p16_pRB | Prone / pro-ferroptotic | 2 | 0.783 | 1.566 | -0.155 | Down | 0.374 | Up | Reduced pro-ferroptotic program | Reduced pro-ferroptotic module despite senescence/SASP activation |
| p53_SAT1_Lipoxygenase_Link | DNA_Damage_Response_DDR  _Telomere | Prone / pro-ferroptotic | 2 | 0.764 | 1.529 | 0.17 | Up | 0.006 | Neutral | Context-dependent association | Pro-ferroptotic module positively associated with senescence |
| Iron_Handling_and_Ferritinophagy | Cell_Cycle_Arrest_p53_p21_p16_pRB | Prone / pro-ferroptotic | 2 | 0.737 | 1.473 | -0.106 | Down | 0.374 | Up | Reduced pro-ferroptotic program | Reduced pro-ferroptotic module despite senescence/SASP activation |
| CoQ10_FSP1_Axis | Cell_Cycle_Arrest_p53_p21_p16_pRB | Protective / resistant | -2 | 0.731 | 1.462 | -0.016 | Neutral | 0.374 | Up | Compensatory protective response | Protective module positively associated with senescence; possible compensatory or context-dependent response |
| Mitochondrial_ROS_and_IronSulfur_Protection | Mitochondrial_Dysfunction_ROS | Protective / resistant | -2 | 0.704 | 1.408 | 0.09 | Up | -0.246 | Down | Context-dependent association | Protective module increased while senescence program is reduced |
| Iron_Handling_and_Ferritinophagy | SASP_GrowthFactors_TGF_Signals | Prone / pro-ferroptotic | 2 | 0.697 | 1.393 | -0.106 | Down | -0.671 | Down | Context-dependent association | Pro-ferroptotic module reduced with senescence program suppression |
| Iron_Handling_and_Ferritinophagy | NFkB_STING_Pathway_Regulators | Prone / pro-ferroptotic | 2 | 0.689 | 1.378 | -0.106 | Down | -0.063 | Down | Context-dependent association | Pro-ferroptotic module reduced with senescence program suppression |
| Iron_Handling_and_Ferritinophagy | SASP_Proteases_ECM_Remodeling | Prone / pro-ferroptotic | 2 | 0.686 | 1.372 | -0.106 | Down | 0.614 | Up | Reduced pro-ferroptotic program | Reduced pro-ferroptotic module despite senescence/SASP activation |
| CoQ10_FSP1_Axis | SASP_GrowthFactors_TGF_Signals | Protective / resistant | -2 | 0.681 | 1.362 | -0.016 | Neutral | -0.671 | Down | Compensatory protective response | Protective module positively associated with senescence; possible compensatory or context-dependent response |
| CoQ10_FSP1_Axis | SASP_Proteases_ECM_Remodeling | Protective / resistant | -2 | 0.677 | 1.354 | -0.016 | Neutral | 0.614 | Up | Compensatory protective response | Protective module positively associated with senescence; possible compensatory or context-dependent response |
| PUFA_Peroxidation_and_Phospholipid_Assembly | SASP_Cytokines_Chemokines | Prone / pro-ferroptotic | 2 | 0.676 | 1.351 | 0.219 | Up | -0.239 | Down | Pro-ferroptotic activation | Pro-ferroptotic module increased while senescence program is reduced |
| CoQ10_FSP1_Axis | Hepatic_Stellate_Cell_Senescence_and_Fibrosis | Protective / resistant | -2 | 0.674 | 1.349 | -0.016 | Neutral | 0.537 | Up | Compensatory protective response | Protective module positively associated with senescence; possible compensatory or context-dependent response |
| Iron_Handling_and_Ferritinophagy | SASP_Cytokines_Chemokines | Prone / pro-ferroptotic | 2 | 0.674 | 1.348 | -0.106 | Down | -0.239 | Down | Context-dependent association | Pro-ferroptotic module reduced with senescence program suppression |
| Iron_Handling_and_Ferritinophagy | Hepatic_Stellate_Cell_Senescence_and_Fibrosis | Prone / pro-ferroptotic | 2 | 0.668 | 1.335 | -0.106 | Down | 0.537 | Up | Reduced pro-ferroptotic program | Reduced pro-ferroptotic module despite senescence/SASP activation |
| GCH1_BH4_Axis | SASP_Proteases_ECM_Remodeling | Protective / resistant | -2 | -0.647 | 1.294 | -0.503 | Down | 0.614 | Up | Loss of protective defense | Loss of protective ferroptosis-defense with senescence/SASP activation |
| PUFA_Peroxidation_and_Phospholipid_Assembly | DNA_Damage_Response_DDR  _Telomere | Prone / pro-ferroptotic | 2 | 0.63 | 1.259 | 0.219 | Up | 0.006 | Neutral | Context-dependent association | Pro-ferroptotic module positively associated with senescence |
| GCH1_BH4_Axis | SASP_GrowthFactors_TGF_Signals | Protective / resistant | -2 | -0.627 | 1.254 | -0.503 | Down | -0.671 | Down | Context-dependent association | Protective module reduced with senescence program suppression; context-dependent |
| CoQ10_FSP1_Axis | SASP_Cytokines_Chemokines | Protective / resistant | -2 | 0.627 | 1.253 | -0.016 | Neutral | -0.239 | Down | Compensatory protective response | Protective module positively associated with senescence; possible compensatory or context-dependent response |
| CoQ10_FSP1_Axis | DNA_Damage_Response_DDR  _Telomere | Protective / resistant | -2 | 0.623 | 1.245 | -0.016 | Neutral | 0.006 | Neutral | Compensatory protective response | Protective module positively associated with senescence; possible compensatory or context-dependent response |
| CoQ10_FSP1_Axis | NFkB_STING_Pathway_Regulators | Protective / resistant | -2 | 0.619 | 1.239 | -0.016 | Neutral | -0.063 | Down | Compensatory protective response | Protective module positively associated with senescence; possible compensatory or context-dependent response |
| Mitochondrial_ROS_and_IronSulfur_Protection | NFkB_STING_Pathway_Regulators | Protective / resistant | -2 | 0.619 | 1.238 | 0.09 | Up | -0.063 | Down | Context-dependent association | Protective module increased while senescence program is reduced |
| GCH1_BH4_Axis | Hepatic_Stellate_Cell_Senescence_and_Fibrosis | Protective / resistant | -2 | -0.619 | 1.238 | -0.503 | Down | 0.537 | Up | Loss of protective defense | Loss of protective ferroptosis-defense with senescence/SASP activation |
| Ether_Lipid_Peroxisome_Sensitization | DNA_Damage_Response_DDR  _Telomere | Prone / pro-ferroptotic | 2 | 0.614 | 1.229 | -0.155 | Down | 0.006 | Neutral | Context-dependent association | Pro-ferroptotic module positively associated with senescence |
| p53_SAT1_Lipoxygenase_Link | NFkB_STING_Pathway_Regulators | Prone / pro-ferroptotic | 2 | 0.585 | 1.169 | 0.17 | Up | -0.063 | Down | Pro-ferroptotic activation | Pro-ferroptotic module increased while senescence program is reduced |
| Mitochondrial_ROS_and_IronSulfur_Protection | DNA_Damage_Response_DDR  _Telomere | Protective / resistant | -2 | 0.573 | 1.147 | 0.09 | Up | 0.006 | Neutral | Compensatory protective response | Protective module positively associated with senescence; possible compensatory or context-dependent response |
| Ether_Lipid_Peroxisome_Sensitization | SASP_Cytokines_Chemokines | Prone / pro-ferroptotic | 2 | 0.553 | 1.107 | -0.155 | Down | -0.239 | Down | Context-dependent association | Pro-ferroptotic module reduced with senescence program suppression |
| GCH1_BH4_Axis | Cell_Cycle_Arrest_p53_p21_p16  _pRB | Protective / resistant | -2 | -0.519 | 1.039 | -0.503 | Down | 0.374 | Up | Loss of protective defense | Loss of protective ferroptosis-defense with senescence/SASP activation |
| Mitochondrial_ROS_and_IronSulfur  _Protection | Cell_Cycle_Arrest_p53_p21_p16  _pRB | Protective / resistant | -2 | 0.486 | 0.971 | 0.09 | Up | 0.374 | Up | Compensatory protective response | Protective module increased with senescence; possible compensatory defense |
| Mitochondrial_ROS_and_IronSulfur  _Protection | Hepatic_Stellate_Cell_Senescence  _and_Fibrosis | Protective / resistant | -2 | 0.478 | 0.956 | 0.09 | Up | 0.537 | Up | Compensatory protective response | Protective module increased with senescence; possible compensatory defense |
| Hepatic_Ferroptosis_Core | SASP_Proteases_ECM_Remodeling | Protective / resistant | -2 | -0.473 | 0.947 | -0.131 | Down | 0.614 | Up | Loss of protective defense | Loss of protective ferroptosis-defense with senescence/SASP activation |
| Hepatic_Ferroptosis_Core | SASP_Cytokines_Chemokines | Protective / resistant | -2 | -0.465 | 0.931 | -0.131 | Down | -0.239 | Down | Context-dependent association | Protective module reduced with senescence program suppression; context-dependent |
| GCH1_BH4_Axis | Mitochondrial_Dysfunction_ROS | Protective / resistant | -2 | 0.463 | 0.926 | -0.503 | Down | -0.246 | Down | Context-dependent association | Protective module reduced with senescence program suppression; context-dependent |
| p53_SAT1_Lipoxygenase_Link | Immune_Clearance_of_Senescent  _Cells | Prone / pro-ferroptotic | 1 | 0.905 | 0.905 | 0.17 | Up | 0.111 | Up | Pro-ferroptotic activation | Pro-ferroptotic module increased with senescence/SASP activation |
| Mitochondrial_ROS_and_IronSulfur  _Protection | SASP_GrowthFactors_TGF_Signals | Protective / resistant | -2 | 0.444 | 0.887 | 0.09 | Up | -0.671 | Down | Context-dependent association | Protective module increased while senescence program is reduced |
| GCH1_BH4_Axis | SASP_Cytokines_Chemokines | Protective / resistant | -2 | -0.438 | 0.875 | -0.503 | Down | -0.239 | Down | Context-dependent association | Protective module reduced with senescence program suppression; context-dependent |
| PUFA_Peroxidation_and_Phospholipid_Assembly | Immune_Clearance_of_Senescent  _Cells | Prone / pro-ferroptotic | 1 | 0.874 | 0.874 | 0.219 | Up | 0.111 | Up | Pro-ferroptotic activation | Pro-ferroptotic module increased with senescence/SASP activation |
| Hepatic_Ferroptosis_Core | Hepatic_Stellate_Cell_Senescence  _and_Fibrosis | Protective / resistant | -2 | -0.436 | 0.871 | -0.131 | Down | 0.537 | Up | Loss of protective defense | Loss of protective ferroptosis-defense with senescence/SASP activation |
| Ether_Lipid_Peroxisome_Sensitization | Immune_Clearance_of_Senescent  _Cells | Prone / pro-ferroptotic | 1 | 0.83 | 0.83 | -0.155 | Down | 0.111 | Up | Reduced pro-ferroptotic program | Reduced pro-ferroptotic module despite senescence/SASP activation |

**Table S4. Ferroptosis–senescence cross-talk in the mouse NASH diet dataset.** Cross-talk between curated liver ferroptosis and senescence modules in diet-induced mouse NASH, including module role, literature direction, correlation, cross-talk strength, module-level expression changes, and dataset-specific interpretation.

| **Ferroptosis Modules** | **Senescence Modules** | **Curated module role** | **Literature direction** | **Correlation** | **CrossTalk Strength** | **Ferro mean log2FC** | **Ferro_direction** | **Senescence mean log2FC** | **Senescence direction** | **Dataset context category** | **Dataset specific interpretation** |
| --- | --- | --- | --- | --- | --- | --- | --- | --- | --- | --- | --- |
| p53_SAT1_Lipoxygenase_Link | Cell_Cycle_Arrest_p53_p21_p16  _pRB | Prone / pro-ferroptotic | 2 | 0.922 | 1.843 | 0.528 | Up | 0.695 | Up | Pro-ferroptotic activation | Pro-ferroptotic module increased with senescence/SASP activation |
| p53_SAT1_Lipoxygenase_Link | Hepatic_Stellate_Cell_Senescence  _and_Fibrosis | Prone / pro-ferroptotic | 2 | 0.92 | 1.84 | 0.528 | Up | 1.208 | Up | Pro-ferroptotic activation | Pro-ferroptotic module increased with senescence/SASP activation |
| p53_SAT1_Lipoxygenase_Link | SASP_Proteases_ECM_Remodeling | Prone / pro-ferroptotic | 2 | 0.918 | 1.836 | 0.528 | Up | 1.685 | Up | Pro-ferroptotic activation | Pro-ferroptotic module increased with senescence/SASP activation |
| PUFA_Peroxidation_and_Phospholipid_Assembly | Hepatic_Stellate_Cell_Senescence  _and_Fibrosis | Prone / pro-ferroptotic | 2 | 0.905 | 1.811 | 0.295 | Up | 1.208 | Up | Pro-ferroptotic activation | Pro-ferroptotic module increased with senescence/SASP activation |
| p53_SAT1_Lipoxygenase  _Link | SASP_GrowthFactors_TGF_Signals | Prone / pro-ferroptotic | 2 | 0.898 | 1.796 | 0.528 | Up | 0.605 | Up | Pro-ferroptotic activation | Pro-ferroptotic module increased with senescence/SASP activation |
| Ether_Lipid_Peroxisome  _Sensitization | Hepatic_Stellate_Cell_Senescence  _and_Fibrosis | Prone / pro-ferroptotic | 2 | 0.892 | 1.784 | 0.173 | Up | 1.208 | Up | Pro-ferroptotic activation | Pro-ferroptotic module increased with senescence/SASP activation |
| PUFA_Peroxidation_and  _Phospholipid_Assembly | SASP_GrowthFactors_TGF_Signals | Prone / pro-ferroptotic | 2 | 0.878 | 1.756 | 0.295 | Up | 0.605 | Up | Pro-ferroptotic activation | Pro-ferroptotic module increased with senescence/SASP activation |
| PUFA_Peroxidation_and  _Phospholipid_Assembly | SASP_Proteases_ECM_Remodeling | Prone / pro-ferroptotic | 2 | 0.874 | 1.748 | 0.295 | Up | 1.685 | Up | Pro-ferroptotic activation | Pro-ferroptotic module increased with senescence/SASP activation |
| Ether_Lipid_Peroxisome  _Sensitization | SASP_GrowthFactors_TGF_Signals | Prone / pro-ferroptotic | 2 | 0.868 | 1.735 | 0.173 | Up | 0.605 | Up | Pro-ferroptotic activation | Pro-ferroptotic module increased with senescence/SASP activation |
| Ether_Lipid_Peroxisome  _Sensitization | SASP_Proteases_ECM_Remodeling | Prone / pro-ferroptotic | 2 | 0.824 | 1.649 | 0.173 | Up | 1.685 | Up | Pro-ferroptotic activation | Pro-ferroptotic module increased with senescence/SASP activation |
| p53_SAT1_Lipoxygenase_Link | SASP_Cytokines_Chemokines | Prone / pro-ferroptotic | 2 | 0.815 | 1.631 | 0.528 | Up | 1.454 | Up | Pro-ferroptotic activation | Pro-ferroptotic module increased with senescence/SASP activation |
| PUFA_Peroxidation_and  _Phospholipid_Assembly | Cell_Cycle_Arrest_p53_p21_p16_pRB | Prone / pro-ferroptotic | 2 | 0.815 | 1.629 | 0.295 | Up | 0.695 | Up | Pro-ferroptotic activation | Pro-ferroptotic module increased with senescence/SASP activation |
| Ether_Lipid_Peroxisome  _Sensitization | Cell_Cycle_Arrest_p53_p21_p16_pRB | Prone / pro-ferroptotic | 2 | 0.783 | 1.566 | 0.173 | Up | 0.695 | Up | Pro-ferroptotic activation | Pro-ferroptotic module increased with senescence/SASP activation |
| p53_SAT1_Lipoxygenase_Link | DNA_Damage_Response_DDR  _Telomere | Prone / pro-ferroptotic | 2 | 0.764 | 1.529 | 0.528 | Up | 0.078 | Up | Pro-ferroptotic activation | Pro-ferroptotic module increased with senescence/SASP activation |
| Iron_Handling_and_Ferritinophagy | Cell_Cycle_Arrest_p53_p21_p16_pRB | Prone / pro-ferroptotic | 2 | 0.737 | 1.473 | 0.067 | Up | 0.695 | Up | Pro-ferroptotic activation | Pro-ferroptotic module increased with senescence/SASP activation |
| CoQ10_FSP1_Axis | Cell_Cycle_Arrest_p53_p21_p16_pRB | Protective / resistant | -2 | 0.731 | 1.462 | 0.027 | Neutral | 0.695 | Up | Compensatory protective response | Protective module positively associated with senescence; possible compensatory or context-dependent response |
| Mitochondrial_ROS_and_IronSulfur_Protection | Mitochondrial_Dysfunction_ROS | Protective / resistant | -2 | 0.704 | 1.408 | -0.074 | Down | -0.196 | Down | Context-dependent association | Protective module reduced with senescence program suppression; context-dependent |
| Iron_Handling_and_Ferritinophagy | SASP_GrowthFactors_TGF_Signals | Prone / pro-ferroptotic | 2 | 0.697 | 1.393 | 0.067 | Up | 0.605 | Up | Pro-ferroptotic activation | Pro-ferroptotic module increased with senescence/SASP activation |
| Iron_Handling_and_Ferritinophagy | NFkB_STING_Pathway_Regulators | Prone / pro-ferroptotic | 2 | 0.689 | 1.378 | 0.067 | Up | -0.058 | Down | Pro-ferroptotic activation | Pro-ferroptotic module increased while senescence program is reduced |
| Iron_Handling_and_Ferritinophagy | SASP_Proteases_ECM_Remodeling | Prone / pro-ferroptotic | 2 | 0.686 | 1.372 | 0.067 | Up | 1.685 | Up | Pro-ferroptotic activation | Pro-ferroptotic module increased with senescence/SASP activation |
| CoQ10_FSP1_Axis | SASP_GrowthFactors_TGF_Signals | Protective / resistant | -2 | 0.681 | 1.362 | 0.027 | Neutral | 0.605 | Up | Compensatory protective response | Protective module positively associated with senescence; possible compensatory or context-dependent response |
| CoQ10_FSP1_Axis | SASP_Proteases_ECM_Remodeling | Protective / resistant | -2 | 0.677 | 1.354 | 0.027 | Neutral | 1.685 | Up | Compensatory protective response | Protective module positively associated with senescence; possible compensatory or context-dependent response |
| PUFA_Peroxidation_and  _Phospholipid_Assembly | SASP_Cytokines_Chemokines | Prone / pro-ferroptotic | 2 | 0.676 | 1.351 | 0.295 | Up | 1.454 | Up | Pro-ferroptotic activation | Pro-ferroptotic module increased with senescence/SASP activation |
| CoQ10_FSP1_Axis | Hepatic_Stellate_Cell_Senescence  _and_Fibrosis | Protective / resistant | -2 | 0.674 | 1.349 | 0.027 | Neutral | 1.208 | Up | Compensatory protective response | Protective module positively associated with senescence; possible compensatory or context-dependent response |
| Iron_Handling_and_Ferritinophagy | SASP_Cytokines_Chemokines | Prone / pro-ferroptotic | 2 | 0.674 | 1.348 | 0.067 | Up | 1.454 | Up | Pro-ferroptotic activation | Pro-ferroptotic module increased with senescence/SASP activation |
| Iron_Handling_and_Ferritinophagy | Hepatic_Stellate_Cell_Senescence  _and_Fibrosis | Prone / pro-ferroptotic | 2 | 0.668 | 1.335 | 0.067 | Up | 1.208 | Up | Pro-ferroptotic activation | Pro-ferroptotic module increased with senescence/SASP activation |
| GCH1_BH4_Axis | SASP_Proteases_ECM_Remodeling | Protective / resistant | -2 | -0.647 | 1.294 | -0.33 | Down | 1.685 | Up | Loss of protective defense | Loss of protective ferroptosis-defense with senescence/SASP activation |
| PUFA_Peroxidation_and_Phospholipid_Assembly | DNA_Damage_Response_DDR  _Telomere | Prone / pro-ferroptotic | 2 | 0.63 | 1.259 | 0.295 | Up | 0.078 | Up | Pro-ferroptotic activation | Pro-ferroptotic module increased with senescence/SASP activation |
| GCH1_BH4_Axis | SASP_GrowthFactors_TGF_Signals | Protective / resistant | -2 | -0.627 | 1.254 | -0.33 | Down | 0.605 | Up | Loss of protective defense | Loss of protective ferroptosis-defense with senescence/SASP activation |
| CoQ10_FSP1_Axis | SASP_Cytokines_Chemokines | Protective / resistant | -2 | 0.627 | 1.253 | 0.027 | Neutral | 1.454 | Up | Compensatory protective response | Protective module positively associated with senescence; possible compensatory or context-dependent response |
| CoQ10_FSP1_Axis | DNA_Damage_Response_DDR  _Telomere | Protective / resistant | -2 | 0.623 | 1.245 | 0.027 | Neutral | 0.078 | Up | Compensatory protective response | Protective module positively associated with senescence; possible compensatory or context-dependent response |
| CoQ10_FSP1_Axis | NFkB_STING_Pathway_Regulators | Protective / resistant | -2 | 0.619 | 1.239 | 0.027 | Neutral | -0.058 | Down | Compensatory protective response | Protective module positively associated with senescence; possible compensatory or context-dependent response |
| Mitochondrial_ROS_and_IronSulfur_Protection | NFkB_STING_Pathway_Regulators | Protective / resistant | -2 | 0.619 | 1.238 | -0.074 | Down | -0.058 | Down | Context-dependent association | Protective module reduced with senescence program suppression; context-dependent |
| GCH1_BH4_Axis | Hepatic_Stellate_Cell_Senescence  _and_Fibrosis | Protective / resistant | -2 | -0.619 | 1.238 | -0.33 | Down | 1.208 | Up | Loss of protective defense | Loss of protective ferroptosis-defense with senescence/SASP activation |
| Ether_Lipid_Peroxisome_Sensitization | DNA_Damage_Response_DDR_Telomere | Prone / pro-ferroptotic | 2 | 0.614 | 1.229 | 0.173 | Up | 0.078 | Up | Pro-ferroptotic activation | Pro-ferroptotic module increased with senescence/SASP activation |
| p53_SAT1_Lipoxygenase_Link | NFkB_STING_Pathway_Regulators | Prone / pro-ferroptotic | 2 | 0.585 | 1.169 | 0.528 | Up | -0.058 | Down | Pro-ferroptotic activation | Pro-ferroptotic module increased while senescence program is reduced |
| Mitochondrial_ROS_and_IronSulfur_Protection | DNA_Damage_Response_DDR  _Telomere | Protective / resistant | -2 | 0.573 | 1.147 | -0.074 | Down | 0.078 | Up | Loss of protective defense | Loss of protective ferroptosis-defense with senescence/SASP activation |
| Ether_Lipid_Peroxisome  _Sensitization | SASP_Cytokines_Chemokines | Prone / pro-ferroptotic | 2 | 0.553 | 1.107 | 0.173 | Up | 1.454 | Up | Pro-ferroptotic activation | Pro-ferroptotic module increased with senescence/SASP activation |
| GCH1_BH4_Axis | Cell_Cycle_Arrest_p53_p21_p16_pRB | Protective / resistant | -2 | -0.519 | 1.039 | -0.33 | Down | 0.695 | Up | Loss of protective defense | Loss of protective ferroptosis-defense with senescence/SASP activation |
| Mitochondrial_ROS_and_IronSulfur_Protection | Cell_Cycle_Arrest_p53_p21_p16_pRB | Protective / resistant | -2 | 0.486 | 0.971 | -0.074 | Down | 0.695 | Up | Loss of protective defense | Loss of protective ferroptosis-defense with senescence/SASP activation |
| Mitochondrial_ROS_and_IronSulfur_Protection | Hepatic_Stellate_Cell_Senescence  _and_Fibrosis | Protective / resistant | -2 | 0.478 | 0.956 | -0.074 | Down | 1.208 | Up | Loss of protective defense | Loss of protective ferroptosis-defense with senescence/SASP activation |
| Hepatic_Ferroptosis_Core | SASP_Proteases_ECM_Remodeling | Protective / resistant | -2 | -0.574 | 1.149 | -0.229 | Down | 1.685 | Up | Loss of protective defense | Loss of protective ferroptosis-defense with senescence/SASP activation |
| Hepatic_Ferroptosis_Core | SASP_Cytokines_Chemokines | Protective / resistant | -2 | -0.582 | 1.163 | -0.229 | Down | 1.454 | Up | Loss of protective defense | Loss of protective ferroptosis-defense with senescence/SASP activation |
| GCH1_BH4_Axis | Mitochondrial_Dysfunction_ROS | Protective / resistant | -2 | 0.463 | 0.926 | -0.33 | Down | -0.196 | Down | Context-dependent association | Protective module reduced with senescence program suppression; context-dependent |
| p53_SAT1_Lipoxygenase_Link | Immune_Clearance_of_Senescent  _Cells | Prone / pro-ferroptotic | 1 | 0.905 | 0.905 | 0.528 | Up | 1.39 | Up | Pro-ferroptotic activation | Pro-ferroptotic module increased with senescence/SASP activation |
| Mitochondrial_ROS_and_IronSulfur_Protection | SASP_GrowthFactors_TGF_Signals | Protective / resistant | -2 | 0.444 | 0.887 | -0.074 | Down | 0.605 | Up | Loss of protective defense | Loss of protective ferroptosis-defense with senescence/SASP activation |
| GCH1_BH4_Axis | SASP_Cytokines_Chemokines | Protective / resistant | -2 | -0.438 | 0.875 | -0.33 | Down | 1.454 | Up | Loss of protective defense | Loss of protective ferroptosis-defense with senescence/SASP activation |
| PUFA_Peroxidation_and_Phospholipid_Assembly | Immune_Clearance_of_Senescent  _Cells | Prone / pro-ferroptotic | 1 | 0.874 | 0.874 | 0.295 | Up | 1.39 | Up | Pro-ferroptotic activation | Pro-ferroptotic module increased with senescence/SASP activation |
| Hepatic_Ferroptosis_Core | Hepatic_Stellate_Cell_Senescence  _and_Fibrosis | Protective / resistant | -2 | -0.436 | 0.871 | 0.097 | Up | 1.208 | Up | Compensatory protective response | Protective module increased with senescence; possible compensatory defense |
| Ether_Lipid_Peroxisome_Sensitization | Immune_Clearance_of_Senescent  _Cells | Prone / pro-ferroptotic | 1 | 0.83 | 0.83 | 0.173 | Up | 1.39 | Up | Pro-ferroptotic activation | Pro-ferroptotic module increased with senescence/SASP activation |
